# Electromagnetic exposure safety assessment of electromagnetic security gates: a review

**DOI:** 10.3389/fpubh.2026.1935419

**Published:** 2026-09-18

**Authors:** Weijiang Bai, Mai Lu

**Affiliations:** Key Laboratory of Opto-Electronic Technology and Intelligent Control of Ministry of Education, Lanzhou Jiaotong University, Lanzhou, China

**Keywords:** electromagnetic exposure, electromagnetic security gates, electronic article surveillance systems, medical devices, radio-frequency identification systems, walk-through metal detectors

## Abstract

In this review, electromagnetic security gates include walk-through metal detectors (WTMDs), electronic article surveillance (EAS) systems, radio-frequency identification (RFID) systems, and millimeter-wave (MMW) security screening systems. These technologies are widely used in public-security screening, theft prevention, access control, and industrial inspection, making electromagnetic-exposure safety a public-health concern. This review traces their technological development, summarizes operating principles and emission characteristics, and examines potential biological effects of electromagnetic-field exposure. It also reviews exposure-assessment methods and applies an adapted evidence-appraisal framework to synthesize experimental, numerical, and epidemiological evidence, evaluate interactions with active medical devices and passive metallic implants, and assess evidence strength and limitations. Particular attention is given to potentially susceptible populations and frequency-specific exposure metrics. Major evidence gaps and future research priorities are identified.

## 1 Introduction

Electromagnetic security gates are widely deployed in airports, railway and metro stations, shopping malls, and schools (1). These systems expose large and heterogeneous populations, including potentially susceptible groups such as children, pregnant women and fetuses, and individuals with medical devices (2–4). Concerns about possible health effects have prompted regulatory attention; the U.S. Food and Drug Administration (FDA), for example, has advised manufacturers to provide labels or signage discouraging users from lingering near or leaning against these systems during passage (5).

Bioelectromagnetic research indicates that electromagnetic fields across different frequency ranges may affect the nervous, immune, and reproductive systems (6, 7). Because security gates span low-frequency, radio-frequency, and millimeter-wave bands, exposure conditions and relevant safety metrics vary across device types (1, 8–10). This heterogeneity complicates health-risk assessment and supports systematic evaluation against established exposure limits.

This review integrates WTMD, EAS, RFID, and MMW security-screening systems within a unified electromagnetic-exposure safety framework. Its distinctive contribution is the integrated synthesis of device characteristics, field measurements, human dosimetry, epidemiological evidence, and interactions with active medical devices and passive metallic implants, with explicit consideration of susceptible populations and frequency-specific exposure metrics. An adapted evidence-appraisal framework evaluates directness, exposure characterization, methodological robustness, consistency and reproducibility, and domain-specific evidence gaps.

## 2 Review methodology

### 2.1 Literature search strategy

A structured literature search was conducted to identify studies relevant to the electromagnetic exposure safety of security gates. PubMed, Web of Science Core Collection, Scopus, and IEEE Xplore were searched, with the literature search last updated in July 2026. No lower publication-year limit was imposed because early studies remain important for tracing the technological development of security gates and the evolution of electromagnetic-exposure assessment.

The search strategy combined three groups of terms. Device-related terms included “walk-through metal detector,” “WTMD,” “electronic article surveillance,” “EAS,” “radio-frequency identification,” “RFID,” “millimeter-wave security screening,” and “electromagnetic security gate.” Exposure-related terms included “electromagnetic exposure,” “electric field,” “magnetic field,” “magnetic flux density,” “dosimetry,” “induced electric field,” “current density,” “specific absorption rate,” “SAR,” “power density,” and “electromagnetic interference.” Additional terms related to health outcomes and potentially susceptible populations included “biological effect,” “health effect,” “occupational exposure,” “worker,” “child,” “pregnancy,” “fetus,” “pacemaker,” “implantable medical device,” and “medical device interference.” Terms within each concept group were combined using the Boolean operator “OR,” whereas device-related terms were combined with exposure- or health-related terms using “AND.” Database-specific syntax was adapted as required.

Official documents from ICNIRP, IEEE, IEC, relevant European standardization bodies, and the U.S. Food and Drug Administration were also reviewed. Reference lists of key reviews and primary studies were screened for additional eligible publications. Commercial market reports were used only for contextual information and were excluded from the scientific evidence appraisal.

### 2.2 Eligibility criteria and study selection

Studies were eligible if they addressed WTMD, EAS, RFID, or MMW screening systems or provided evidence directly relevant to their electromagnetic-exposure assessment. Eligible evidence included field measurements, numerical dosimetry, biological and health-effect studies, occupational or population-based epidemiology, medical-device interference studies, and relevant systematic reviews or meta-analyses. Exposure guidelines, technical standards, and regulatory documents were included when they defined exposure restrictions, measurement procedures, or compliance requirements.

Historical and technical studies were retained when needed to establish device development, operating principles, emission characteristics, or exposure-relevant configurations. Conference papers were included when they provided original technical or dosimetric information not adequately reported elsewhere.

Studies were excluded if they were unrelated to electromagnetic exposure or safety; focused only on detection algorithms, image reconstruction, target recognition, or security performance without exposure-relevant information; duplicated a more complete report; or used models or exposure conditions with insufficient relevance to the interaction mechanisms or human-exposure scenarios considered here. After duplicate removal, titles and abstracts were screened, followed by full-text assessment. Additional records identified from reference lists were assessed using the same criteria.

### 2.3 Data extraction and evidence appraisal

For each eligible study, extracted information included security-gate type, operating frequency, source configuration and waveform, exposure scenario, study design, population or anatomical model, assessment method, exposure metric, comparison with applicable guidelines, principal findings, and reported limitations. Medical-device studies were additionally characterized by device type, operating mode, exposure configuration, and interference endpoint; epidemiological studies by population, sample size, exposure assessment, health outcome, follow-up, statistical power where reported, and major sources of bias or uncertainty.

Because the evidence base spans field measurements, numerical simulations, biological experiments, epidemiology, and medical-device interference studies, an adapted structured framework was used to appraise evidence consistently across domains. Four dimensions were considered: (1) directness to actual security-gate exposure; (2) exposure characterization, including frequency, waveform, field strength, distance, geometry, duration, and dosimetry; (3) methodological robustness, including sample size where relevant, calibration, model resolution, validation, uncertainty analysis, and appropriateness of comparison criteria; and (4) consistency and reproducibility across independent studies.

Overall evidence strength within each domain was categorized as high, moderate, low, or very low. High-strength evidence was direct, well characterized, methodologically robust, and consistent across independent studies. Moderate evidence remained limited by factors such as sample size, exposure characterization, validation, or replication. Low evidence had substantial methodological limitations, indirect exposure conditions, or limited consistency, whereas very-low evidence relied mainly on highly indirect models, isolated observations, poorly characterized exposure, or findings with substantial uncertainty.

Ratings were assigned within each evidence domain rather than used to rank fundamentally different study types. Because device types, frequencies, exposure metrics, populations, configurations, and endpoints were highly heterogeneous, a single quantitative synthesis was not appropriate. Evidence was therefore synthesized narratively and comparatively by device category, study design, exposure characteristics, and evidence strength. Domain-level ratings are reported in the relevant synthesis sections and summarized in Table 1.

**Table 1:** Structured appraisal of evidence strength across major evidence domains relevant to electromagnetic security-gate exposure.

Evidence domain	Directness	Exposure characterization	Methodological robustness	Consistency and reproducibility	Overall evidence strength
Security-gate-specific epidemiology	Direct, mainly occupational EAS	Individual exposure incompletely characterized; non-uniform and intermittent	Few health-outcome studies; small samples; no long-term gate-specific cohorts	Limited independent evidence	Low
Broader ELF/RF epidemiology	Indirect	Exposure sources and patterns differ from security gates	Cohort, pooled, systematic-review, and meta-analytic evidence; misclassification and confounding remain	Moderate for several outcomes; variable across endpoints	Moderate
Experimental field characterization	Direct	Frequency, waveform, distance, and spatial fields generally characterized; protocols vary	Actual systems measured; instrumentation and sampling not fully standardized	Spatial trends generally consistent	Moderate
Experimental interactions with active medical devices	Direct and clinically relevant	Distance, duration, waveform, and device configuration reported but heterogeneous	In vivo, bench, simulator, and clinical evidence; devices and protocols vary	Broadly consistent after accounting for exposure conditions	Moderate
Human numerical dosimetry	Direct or highly relevant	Internal dose quantified; sensitive to source, anatomy, orientation, and tissue properties	High-resolution models available; experimental validation incomplete	General trends consistent; quantitative values vary	Moderate
Numerical/analytical interactions with medical devices and implants	Direct or highly relevant	Coupling quantified; simplified geometries and worst-case assumptions common	Mechanistically informative; few device models and independent validations	Limited independent replication	Low

Evidence-strength ratings refer to the overall body of evidence within each domain and should not be interpreted as quality ratings for individual studies. Ratings were assigned according to the structured appraisal framework described in Section 2.3, considering directness, exposure characterization, methodological robustness, and consistency and reproducibility.

## 3 Historical development and market trends of electromagnetic security gates

### 3.1 Historical development and current status of electromagnetic security gates

In this review, electromagnetic security gates include walk-through metal detectors (WTMDs), electronic article surveillance (EAS) systems, radio-frequency identification (RFID) systems, and millimeter-wave (MMW) security screening systems (11).

Metal-detection gates date back to 1925, when the technology was initially used for mineral exploration (12). The first commercial WTMD was introduced in 1960 and was used mainly in mining and industrial settings to prevent theft of valuable minerals. In 1970, metal detectors were formally introduced into security-screening applications. Following the terrorist attacks of September 11, 2001, security requirements in densely populated public spaces became more stringent, accelerating the adoption of WTMDs as standard screening equipment.

EAS technology emerged in the 1960s, initially for theft prevention in libraries and retail stores (13–15). MW, RF, EM, and AM technologies subsequently entered commercial use, progressively extending EAS applications beyond conventional retail theft prevention (8, 16). The corresponding timeline is shown in Figure 1.

**Figure 1:**
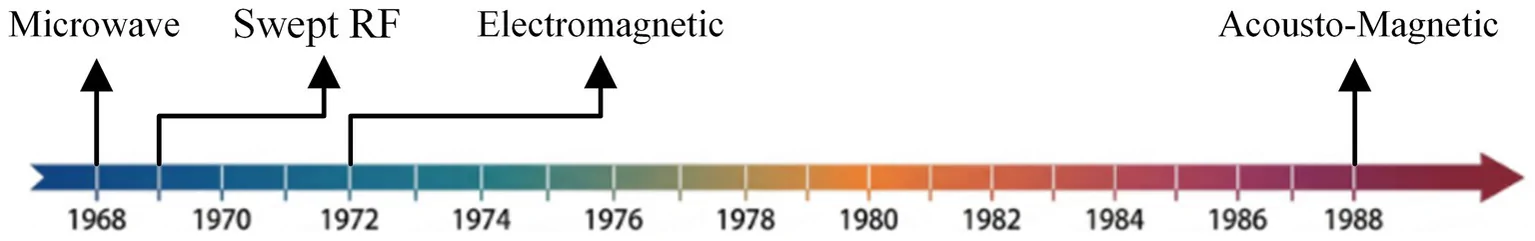
Timeline of the commercial introduction of major EAS technologies.

RFID technology can be traced to the 1950s, when early systems evolved from radar-based identification technologies (17). LF RFID matured during the 1970s; although its reading range was limited, it showed relatively strong resistance to interference in complex electromagnetic environments. HF RFID emerged in the 1980s with improved reading distance and data-transmission rates. UHF RFID gained prominence in the 1990s, and reading ranges exceeding 10 m together with declining tag costs supported large-scale deployment. Since the beginning of the twenty-first century, RFID has been widely adopted (9).

Research on MMW personnel-screening technology began in the 1990s (10). Early work was conducted mainly by U.S. national laboratories and affiliated research institutions, with applications concentrated in military and other high-security settings. Because of limitations in available components and signal-processing systems, early devices relied predominantly on passive MMW imaging (18). After 2005, improvements in imaging speed, spatial resolution, and signal-to-noise ratio accelerated the adoption of active MMW systems. Their ability to detect non-metallic threats further supported deployment in public-security environments (19).

### 3.2 Market landscape and development trends of electromagnetic security gates

Because primary global deployment data for security gates remain limited, commercial market-research reports were used as supplementary sources. Based on segmented data from these reports (20–23), Figure 2 summarizes regional market shares, technology-specific shares, and compound annual growth rates (CAGRs) as of 2025.

**Figure 2:**
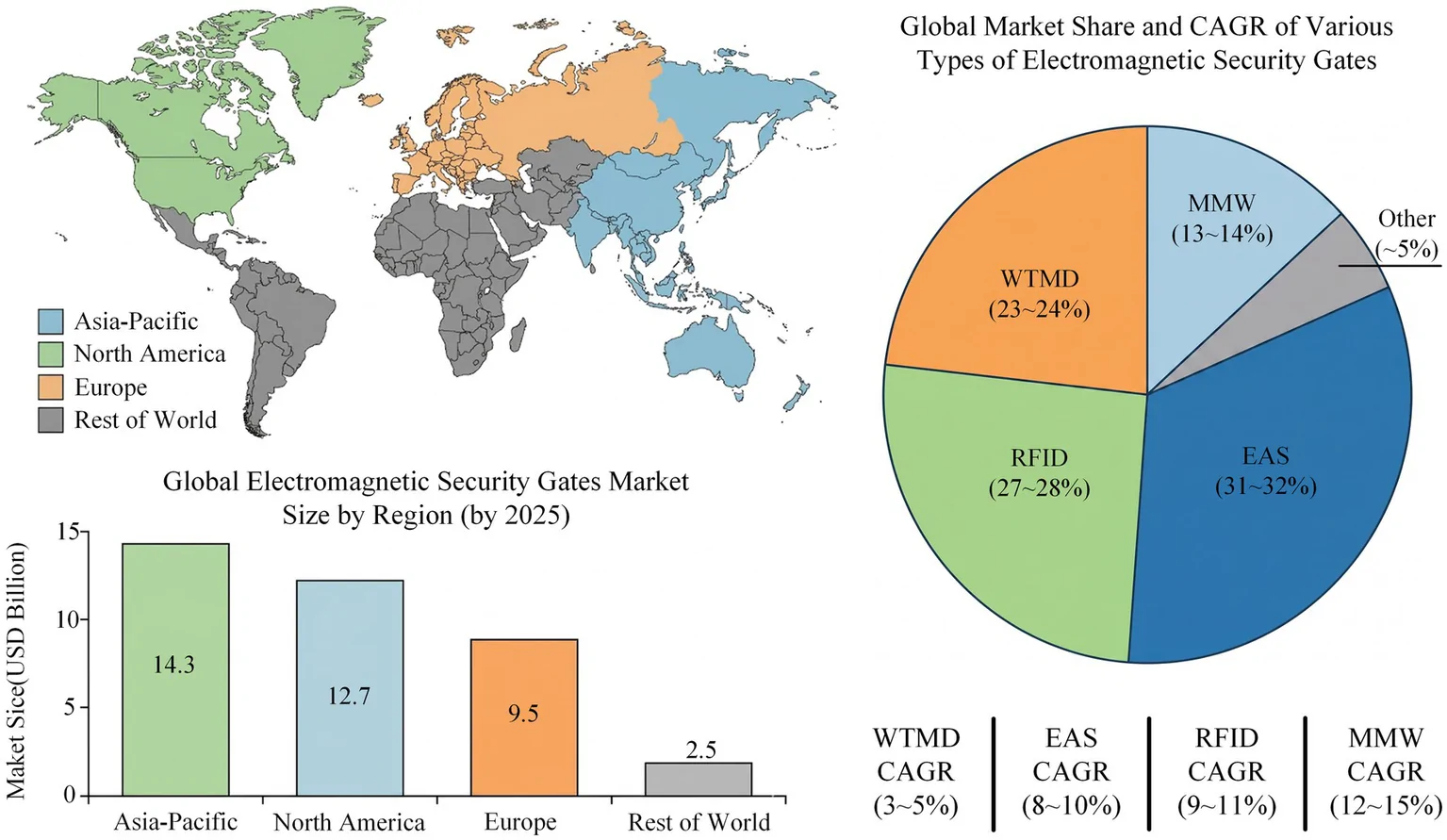
Regional market shares, technology-specific market shares, and compound annual growth rates of the global market for electromagnetic security gates as of 2025 [Compiled from Markets and Markets (20, 21), Data Bridge Market Research (22), and Persistence Market Research (23)].

The WTMD market is driven mainly by equipment replacement and the installation of basic security infrastructure in emerging markets. EAS systems remain important for retail loss prevention. RFID systems are increasingly used in traceability, security screening, and intelligent management, while MMW systems are expanding in airports and major transportation hubs. Emerging technologies such as terahertz screening remain constrained by relatively high cost (20–23).

## 4 Bioelectromagnetics

### 4.1 Current research landscape and significance of bioelectromagnetics

Bioelectromagnetics encompasses endogenous electromagnetic activity in biological systems, interactions between externally applied electromagnetic fields and biological tissues, and the application of electromagnetic phenomena in medicine and the life sciences (24, 25). Neural, cardiac, and muscular activity generates measurable electromagnetic signals that underpin a range of bioelectromagnetic measurements and biomedical applications (24, 25).

Research spans a broad range of frequencies and levels of biological organization. Investigations have expanded from whole-body and organ-level exposure to cellular, molecular, and computational approaches, while addressing both biomedical applications and potential adverse effects (26–29). Advances in anatomical modeling, electromagnetic dosimetry, and computational exposure assessment have also improved characterization of internal fields under complex exposure conditions (26, 30).

Humans are continuously exposed to electromagnetic fields from natural and anthropogenic sources, including power systems, communication technologies, medical equipment, industrial devices, and security-screening systems (31). Biological interaction depends on frequency, field strength, waveform, exposure duration, spatial distribution, body geometry, and tissue properties. In particular, tissue permittivity and electrical conductivity vary substantially across tissue types and frequencies (32–34). The external field, the internal dose produced within the body, and the subsequent biological response should therefore be considered distinct but interconnected components of exposure assessment (24, 30, 31).

### 4.2 Mechanisms of electromagnetic-field interactions with biological systems

The interaction of external electromagnetic fields with biological systems is governed by coupling with conductive and dielectric tissues (6, 7). When an external field encounters the body, its distribution is modified by induction, reflection, transmission, and absorption, whose relative importance depends on frequency. Tissue conductivity and permittivity, anatomy, body orientation, source distance, and spatial field distribution further influence the magnitude and localization of internal fields (6, 7). Consequently, identical external field strengths do not necessarily produce identical internal exposure in different tissues or individuals. A measurable biological response may follow the initial physical interaction, but it does not by itself indicate an adverse health effect (35, 36).

At low and intermediate frequencies, time-varying electric and magnetic fields interact with the body mainly by inducing internal electric fields and currents (6, 7). Their magnitude depends on the temporal variation of the external field, tissue conductivity, body dimensions, and exposure geometry. When internal electric fields become sufficiently strong, they may stimulate electrically excitable tissues, including peripheral nerves and muscles. This mechanism forms an established biophysical basis for low-frequency exposure restrictions. The intermediate-frequency range is a transition region in which induced-field effects remain relevant while electromagnetic-energy absorption becomes increasingly important (6, 7). Studies in this range have examined neurological, developmental, reproductive, cellular, and other endpoints, but the evidence remains heterogeneous because of differences in exposure systems, dosimetry, and experimental design (37).

At radiofrequencies, electromagnetic energy is increasingly absorbed by biological tissues and converted into heat through dielectric and conductive losses (6, 7, 38). The magnitude and distribution of absorption depend on frequency, tissue properties, body geometry, polarization, and exposure duration. Specific absorption rate (SAR) is therefore widely used to characterize radiofrequency energy absorption within the body. As frequency increases, penetration depth decreases and energy deposition becomes progressively more superficial. In the millimeter-wave range, interaction occurs predominantly near the body surface, particularly in the skin, and power-density-based quantities become increasingly important for exposure assessment (7, 38). The dominant interaction mechanism therefore changes progressively with frequency rather than at a single sharp boundary.

Potential non-thermal responses involving oxidative stress, calcium regulation, membrane processes, and cellular signaling have also been investigated (35, 36, 39). However, their reproducibility and biological significance remain uncertain and depend strongly on exposure conditions and experimental models (38, 39). Evidence linking RF-EMF exposure to oxidative-stress biomarkers remains inconsistent and is affected by limitations in exposure characterization and experimental quality (39). Resonance-related interactions, magnetosensitive molecular processes, and other weak-field mechanisms have also been proposed, but their contribution to adverse effects under typical environmental exposure conditions has not been established (24).

These frequency-dependent mechanisms provide the biological basis for assessing electromagnetic security gates (6, 7, 35, 36, 38, 39). WTMDs and low-frequency EAS systems are assessed mainly using induced-field metrics, whereas RF EAS and RFID systems require greater consideration of electromagnetic-energy absorption. MMW screening systems primarily produce superficial exposure. Accordingly, frequency-specific metrics should be used: induced electric fields and current densities are relevant to electrostimulation at low and intermediate frequencies, SAR characterizes radiofrequency absorption, and power density is more appropriate for superficial millimeter-wave exposure (6, 7, 38). This distinction underpins the limit-based comparisons presented below.

### 4.3 Potential biological and health effects of electromagnetic fields

At the molecular and cellular levels, EMF exposure has been investigated in relation to oxidative stress, genotoxicity, cellular proliferation and apoptosis, membrane function, intracellular signaling, and calcium homeostasis (40, 41). At the organ-system level, studies have examined possible effects on the nervous, endocrine, cardiovascular, immune, reproductive, and sensory systems (42, 43). Reported responses include changes in neural activity, neuroendocrine regulation, cardiovascular parameters, reproductive function, and sensory responses (42, 43). However, their consistency, exposure dependence, reversibility, and clinical significance vary substantially among studies (44, 45). Biological effects observed under specific experimental conditions should therefore not be interpreted directly as evidence of adverse effects in humans, particularly when exposure conditions differ from those encountered in everyday environments (44, 45).

Some individuals report non-specific symptoms such as headache, fatigue, dizziness, sleep disturbance, anxiety, skin sensations, or impaired concentration that they attribute to EMF exposure, commonly described as electromagnetic hypersensitivity (EHS) or idiopathic environmental intolerance attributed to electromagnetic fields (46). Controlled studies have not established a consistent causal relationship between these symptoms and EMF exposure (47, 48). In security-gate environments, a separate and more directly assessable concern is electromagnetic interference with active medical devices. Because this involves interaction between an external field and electronic equipment rather than a direct biological effect, it is addressed separately in later sections.

### 4.4 Epidemiological evidence

Potential long-term health effects of EMF exposure have also been investigated epidemiologically. Extremely low-frequency magnetic fields and radiofrequency electromagnetic fields have been classified by the International Agency for Research on Cancer as possibly carcinogenic to humans (Group 2B) (49–51). This classification reflects limited evidence of carcinogenicity rather than a quantitative estimate of risk at a specific environmental exposure level. Associations with childhood leukemia, selected brain tumors, and other cancers have been studied extensively, but the strength and consistency of the evidence vary with frequency range, exposure source, study period, and exposure-assessment method (49–52).

Direct epidemiological evidence specific to security-gate exposure remains limited. Occupational studies have focused mainly on personnel working near EAS systems, including cashiers and library workers, because their exposure may be repeated or prolonged compared with the transient exposure of the general public. Exposure patterns depend strongly on workplace layout, distance from the gate, frequency of passage, and other electromagnetic sources in the surrounding environment (2, 53, 54). Health-outcome evidence is much less developed. A pilot study of women with EAS-related occupational exposure found no consistent increase in adverse reproductive outcomes; however, its limited sample size and uncertainty in exposure classification preclude firm conclusions (55).

For long-term ELF magnetic-field exposure, childhood leukemia remains the most extensively investigated outcome (49–52). Recent pooled and meta-analytic evidence has not consistently reproduced associations reported in earlier datasets, leaving uncertainty about the overall relationship between long-term magnetic-field exposure and childhood leukemia (56, 57). Occupational ELF studies have likewise produced mixed findings. Large occupational cohorts and updated meta-analyses do not provide consistent evidence of an overall increase in hematolymphopoietic cancer risk, although associations for selected outcomes and highly exposed subgroups cannot be completely excluded (58).

Long-term RF epidemiology includes cohort, case–control, and occupational studies (52, 59–61). Prospective cohort evidence has not shown a consistent association between cumulative mobile-phone exposure and the major brain tumors examined. Systematic reviews and meta-analyses of observational studies likewise generally do not indicate increased risks for several extensively studied cancers under the RF exposure conditions evaluated (52). Evidence is not equally robust for all exposure sources and outcomes, however, and occupational RF studies are particularly limited by imprecise exposure characterization. These broader findings provide useful context but cannot be directly extrapolated to RF EAS, RFID, or MMW security systems.

Several methodological limitations constrain causal inference in EMF epidemiology (52). Exposure is often reconstructed from occupational titles, residential distance, self-reported device use, or limited field measurements, creating potential misclassification. Technological change, confounding, selection and recall bias, and limited statistical power further complicate interpretation. These limitations are especially relevant to security-gate exposure because it is spatially non-uniform and intermittent.

Within the structured appraisal framework used in this review, direct epidemiological evidence specific to security-gate exposure was rated as low in strength because of the limited number of health-outcome studies, uncertainty in individual exposure assessment, and the absence of long-term security-gate-specific cohorts. Broader ELF and RF epidemiological evidence was rated as moderate for contextual interpretation because it includes cohort studies, pooled analyses, systematic reviews, and meta-analyses; however, differences in exposure sources and patterns limit direct extrapolation to security-gate environments. Epidemiological findings should therefore be interpreted together with experimental, dosimetric, and mechanistic evidence and in relation to established exposure guidelines and standards.

### 4.5 Electromagnetic exposure guidelines and standards

Electromagnetic exposure guidelines and standards provide the fundamental framework for protecting individuals from established adverse effects associated with electromagnetic-field exposure. Two major frameworks have been developed around the recommendations of the Institute of Electrical and Electronics Engineers (IEEE) and the International Commission on Non-Ionizing Radiation Protection (ICNIRP), collectively covering frequencies from 0 Hz to 300 GHz and supporting standardized assessments across a wide range of exposure scenarios. Comparative analyses indicate that ICNIRP and IEEE may yield different compliance conclusions, particularly for localized low-frequency exposure, because their basic restrictions and reference levels are not identical (62).

IEEE standards. IEEE Std C95.1-1991 (63) specified safety levels for human exposure to radio-frequency electromagnetic fields over the frequency range of 3 kHz to 300 GHz. The 1999 edition refined the calculation and application of power-density limits (64). IEEE Std C95.6-2002 subsequently established safety levels for low-frequency electromagnetic-field exposure from 0 to 3 kHz (65). IEEE Std C95.1-2005 further incorporated the concept of action levels into the exposure-assessment framework (66). More recently, IEEE Std C95.1-2019 consolidated the assessment of electric, magnetic, and electromagnetic fields into an integrated framework covering the full frequency range from 0 Hz to 300 GHz (67).

ICNIRP guidelines. The ICNIRP guidelines published in 1998 addressed exposure to time-varying electric, magnetic, and electromagnetic fields at frequencies up to 300 GHz and established a unified framework for exposure restrictions (68). In 2010, ICNIRP issued revised guidelines specifically addressing low-frequency electric and magnetic fields from 1 Hz to 100 kHz (69). The 2020 guidelines updated the radiofrequency exposure framework for the range of 100 kHz to 300 GHz, refining the dosimetric approach and providing more detailed restrictions for localized exposure (70).

Given the highly non-uniform near-field exposure generated by electromagnetic security gates (71), international organizations have developed application-specific safety-assessment frameworks. In 2004, ICNIRP issued a statement addressing security and similar devices that use electromagnetic fields, emphasizing the need to assess compliance with basic restrictions, including internally induced current density and specific absorption rate (SAR), under non-uniform exposure conditions (72).

For security-gate-specific assessment, IEC 62369-1 provides standardized procedures for evaluating human exposure from EAS, RFID, and similar short-range devices, including staged compliance assessment under non-uniform fields (73). EN 50364:2018 further specifies product-level human-exposure compliance requirements for these devices over 0 Hz–300 GHz (74).

### 4.6 Regulatory implementation, risk communication, and practical recommendations

Regulatory implementation varies across jurisdictions in legal status, responsible authorities, and compliance mechanisms. In Europe, Council Recommendation 1999/519/EC provides a common framework for general-public exposure, whereas Directive 2013/35/EU establishes minimum legally binding requirements for occupational exposure, including consideration of pregnant workers and workers with implanted medical devices (75, 76). Security-gate-specific technical standards described in Section 4.5 are applied within this broader framework.

In North America, implementation is more decentralized. IEEE C95.1 provides a technical exposure framework in the United States, applicable RF-exposure requirements are enforced through FCC regulations, and medical-device electromagnetic compatibility is addressed separately by FDA guidance (67, 77, 78). Canada applies Health Canada Safety Code 6 through ISED equipment-compliance requirements (79, 80). In the Asia-Pacific region, Australia applies ARPANSA RPS S-1 for RF exposure, whereas China implements the mandatory GB 8702-2014 standard for public electromagnetic-environment exposure (70, 81, 82). These examples show how broadly harmonized scientific principles are implemented through different regulatory mechanisms.

Risk communication should distinguish compliance with exposure limits from the probability of adverse health effects and should acknowledge scientific uncertainty and differences among exposure scenarios (83). Routine passage should be distinguished from prolonged close-range exposure, and exceedance of an external reference level should not automatically be interpreted as exceedance of an internal basic restriction (53). Facility operators should periodically assess compliance, limit prolonged staff occupancy in high-field near-panel areas, reassess exposure after substantial changes in device configuration or site layout, and provide clear precautions or alternative screening for individuals with active implantable medical devices when appropriate (84–100).

Public-health authorities should promote consistent signage and communication for susceptible groups, using proportionate measures that reduce unnecessary exposure without compromising essential security screening. The following checklist summarizes practical considerations for facility operators and public-health practitioners:

1 Identify the security-gate technology and operating frequency, and determine the applicable exposure guideline or regulatory framework.
2 Verify compliance using frequency-appropriate metrics, distinguishing external reference levels from internal basic restrictions where applicable.
3 Prioritize realistic high-exposure scenarios, particularly prolonged close-range occupancy near transmitting panels and repeated occupational exposure, rather than routine passage alone.
4 Identify potentially susceptible individuals, including persons with active implantable medical devices and pregnant workers, and provide appropriate signage, precautions, or alternative screening when indicated.
5 Reassess exposure after substantial changes in equipment configuration, operating parameters, or site layout, and maintain records of compliance assessment and equipment maintenance.
6 Communicate measured or calculated exposure relative to applicable limits without interpreting compliance margins as direct probabilities of adverse health effects.

## 5 Operating principles of electromagnetic security gates

### 5.1 Walk-through metal detectors

WTMDs operate on electromagnetic induction, typically at frequencies of 1–10 kHz (101). A signal generator drives the transmitter coil to produce an alternating magnetic field within the passageway (102). When a metallic object enters the detection zone, induced eddy currents generate a secondary magnetic field that perturbs the primary field. The receiver coil detects this perturbation and converts it into a voltage signal, which is amplified, filtered, and analyzed. An alarm is triggered when a predefined threshold is exceeded (103–105). The operating principle is illustrated in Figure 3.

**Figure 3:**
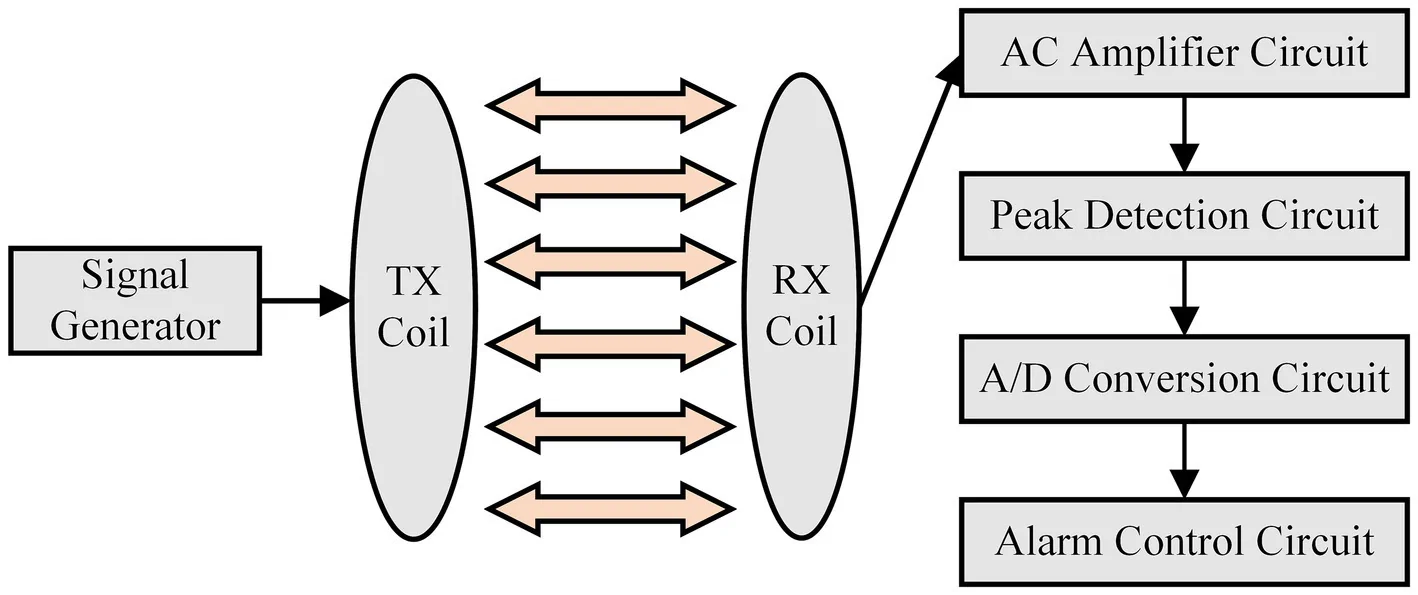
Typical system architecture of a WTMD.

A typical WTMD comprises a gate frame, coil assembly, control unit, alarm unit, power supply, and calibration module. The passageway is generally at least 200 cm high, 70 cm wide, and 50 cm deep. The coil assembly contains transmitter, receiver, and compensation coils (106, 107), with the transmitter coil constituting the principal electromagnetic-emission source. Figure 4 shows a representative six-zone WTMD and its coil arrangement.

**Figure 4:**
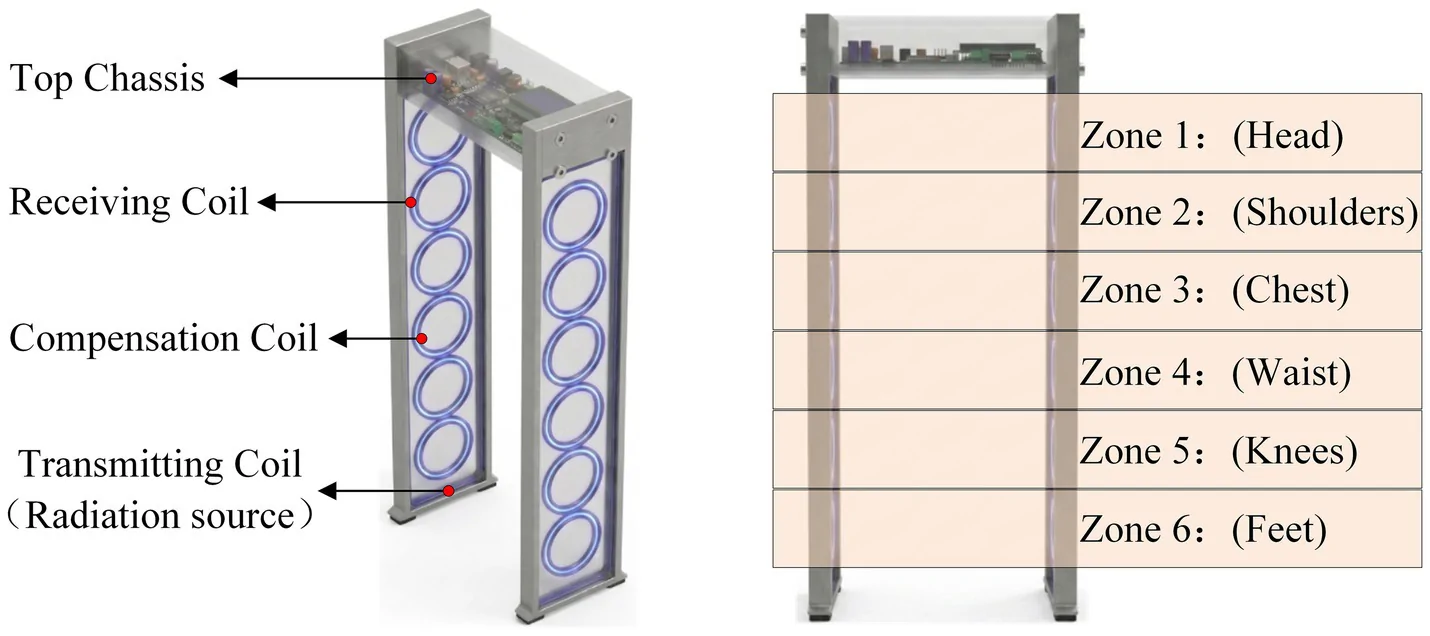
Representative walk-through metal detector and schematic distribution of its coil system.

### 5.2 Electronic article surveillance systems

EAS systems are classified into four principal categories: electromagnetic (EM), acousto-magnetic (AM), radio-frequency (RF), and microwave (MW) systems. Their representative technical characteristics are summarized in Table 2.

**Table 2:** Technical characteristics of major EAS technologies.

System type	Frequency range	Principal tag component	Principal field component
EM EAS	20 Hz–18 kHz	Magnetic strip or wire	Magnetic field
AM EAS	58–132 kHz	Resonant magnetostrictive element	Magnetic field
RF EAS	1.8–10 MHz	Resonant LC circuit	Magnetic field
MW EAS	902–928 MHz and 2,400–2,500 MHz	Diode	Electromagnetic field

EM systems. EM EAS systems use magnetic-harmonic detection. A continuous excitation field drives the magnetic strip of a tag into nonlinear magnetization and saturation, generating harmonic components in the field waveform. The receiver detects these components and triggers an alarm when their amplitude exceeds a predefined threshold (8).

AM systems. AM tags contain a magnetoelastic element that resonates mechanically when excited by a pulsed magnetic field at a specific frequency. After each excitation pulse, the transmitted field decays, allowing the receiver to detect the characteristic ring-down response. An alarm is activated when the signal amplitude and decay profile meet predefined criteria (8, 15).

RF systems. RF EAS systems use resonant-frequency detection. The transmitter sweeps across 1.8–10 MHz to cover the tag resonance. When the tag enters the interrogation zone, its inductor–capacitor (LC) circuit produces a resonant response that may appear as a phase shift, energy absorption, or local-field perturbation. The receiver detects this change and triggers an alarm (8).

MW systems. MW EAS tags typically contain a nonlinear diode element. Microwave interrogation causes the tag to generate and reradiate a response, commonly at an integer harmonic of the incident frequency, which is detected by the receiving antenna (8).

The four operating principles are illustrated in Figure 5.

**Figure 5:**
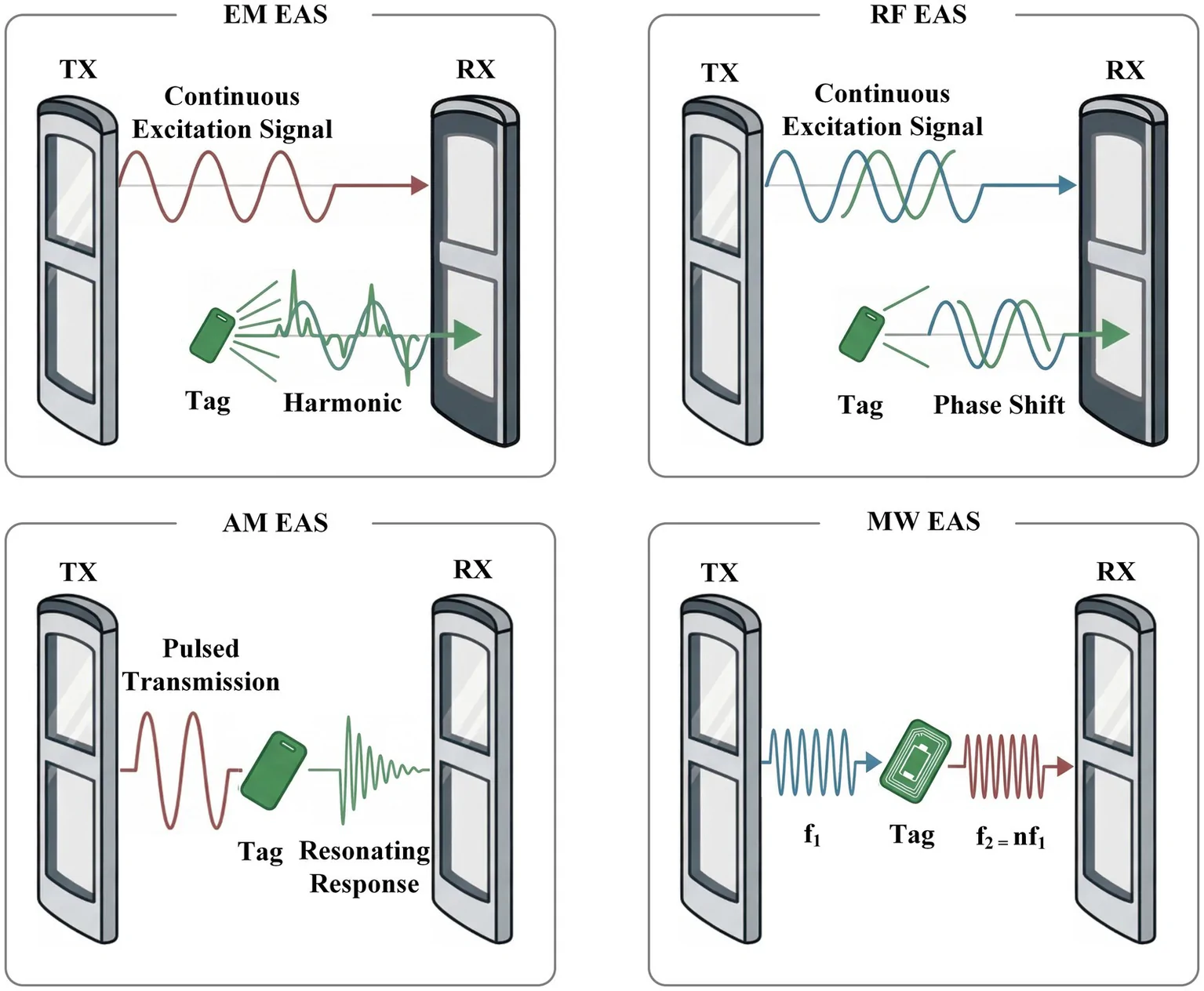
Operating principles of the principal EAS technologies.

EAS transmitting antennas, the principal electromagnetic-emission sources, are commonly implemented as multiturn rectangular loops embedded in the gate panels or as overlapping loops forming an approximately rectangular configuration. Transmission may occur from one or both sides of the passageway. Receiving antennas often use figure-eight or E-shaped configurations to suppress direct coupling from the transmitter (108), as illustrated in Figure 6. Typical gate panels are 150–200 cm high, 20–80 cm wide, and 2–10 cm thick, with passageway widths of approximately 80–200 cm (16, 71).

**Figure 6:**
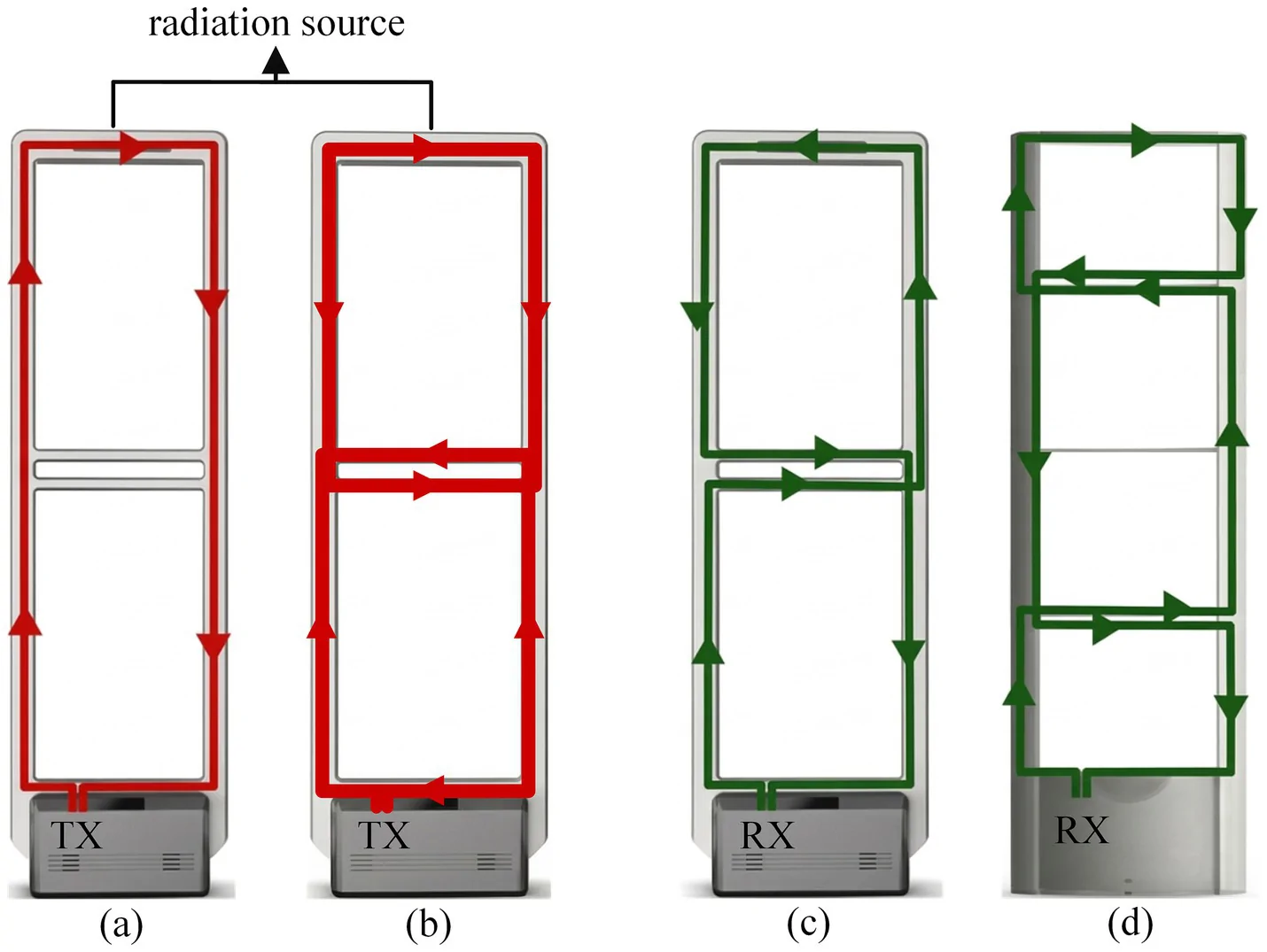
Representative antenna configurations embedded within the gate panels of EAS systems: **(a,b)** transmitting (TX) antenna configurations; **(c,d)** receiving (RX) antenna configurations.

### 5.3 Radio-frequency identification systems

Table 3 summarizes representative RFID frequency ranges and technical characteristics (109). An RFID system generally comprises a reader, one or more antennas, RFID tags, and a backend processing unit, as illustrated in Figure 7. Antenna architecture depends on operating frequency: LF and HF systems commonly use inductively coupled loop antennas similar to those of certain EAS systems, whereas UHF RFID gates typically employ microstrip patch antennas, dipole arrays, or planar directional antennas within the gate panels (9).

**Table 3:** Classification and technical characteristics of RFID systems.

Frequency band	Representative frequency range	Principal characteristics	Common tag types
Low frequency (LF)	30–300 kHz; commonly 125–134 kHz	Short reading range; relatively low read rate; comparatively robust performance near liquids and metals	Primarily passive
High frequency (HF)	3–30 MHz; commonly 13.56 MHz	Short-to-medium reading range; moderate read rate	Primarily passive
Ultra-high frequency (UHF)	860–960 MHz	Longer reading range; higher read rate; greater sensitivity to environmental conditions	Passive, semi-passive, or active
Microwave (MW)	Commonly 2.45 GHz	Longer reading range and higher data-transfer rate; greater sensitivity to propagation conditions	Passive or active

**Figure 7:**
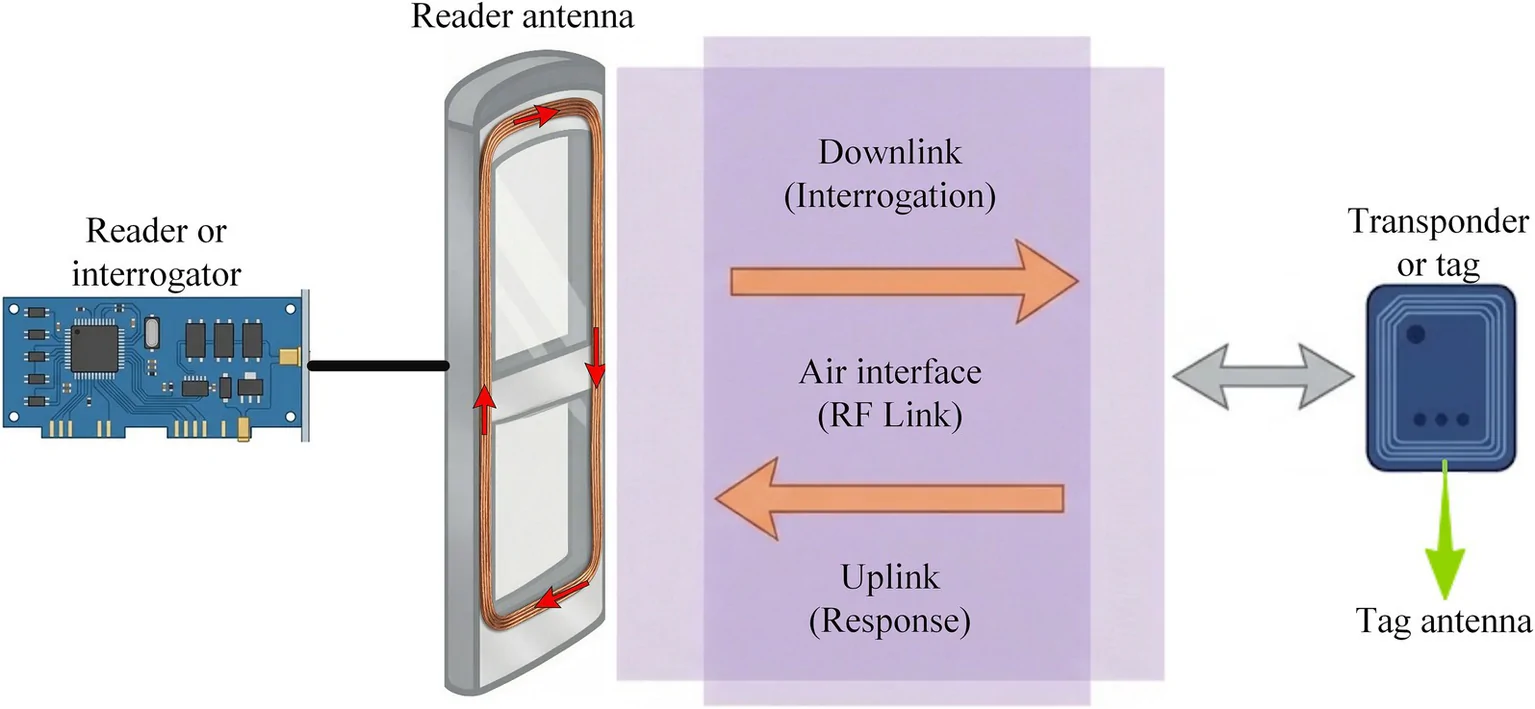
Operating principle of an RFID system.

Passive RFID tags harvest energy from the reader’s interrogation signal and return stored data through modulation. Active tags use an internal power source and can support longer transmission ranges. The backend processor decodes the received data, verifies tag identity, and executes the required control procedures (110).

A typical RFID gate is approximately 150 cm high, 60 cm wide, and 4 cm thick. Passageway widths generally range from 60 to 160 cm, with approximately 120 cm commonly used (111, 112).

### 5.4 Millimeter-wave security screening systems

MMW screening systems typically operate at 30–300 GHz and are classified as passive or active according to their transmission and reception mechanisms (113–115), as illustrated in Figure 8. They generally comprise antenna arrays, signal-processing modules, image-reconstruction algorithms, and display units.

**Figure 8:**
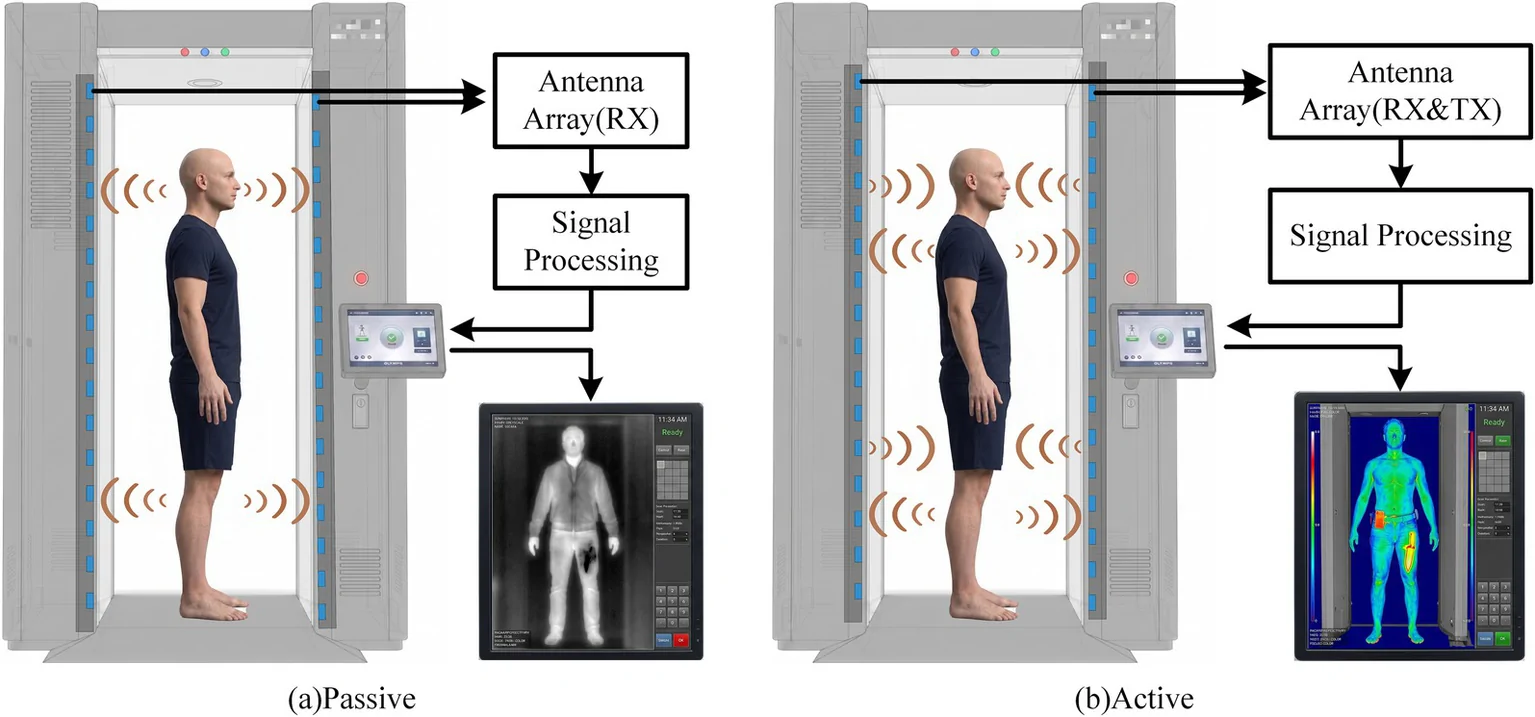
Operating principles of MMW security screening systems: **(a)** passive system and **(b)** active system.

In gate-based configurations, transmitting (Tx) and receiving (Rx) arrays are integrated into panels on both sides of the passageway. One-dimensional linear or two-dimensional planar arrays are commonly used. Depending on the required imaging performance, hardware complexity, and computational cost, systems may employ massive multiple-input multiple-output (MIMO) arrays with hundreds or thousands of elements or sparse arrays with orthogonal or parallel arrangements (116–118), as shown in Figure 9.

**Figure 9:**
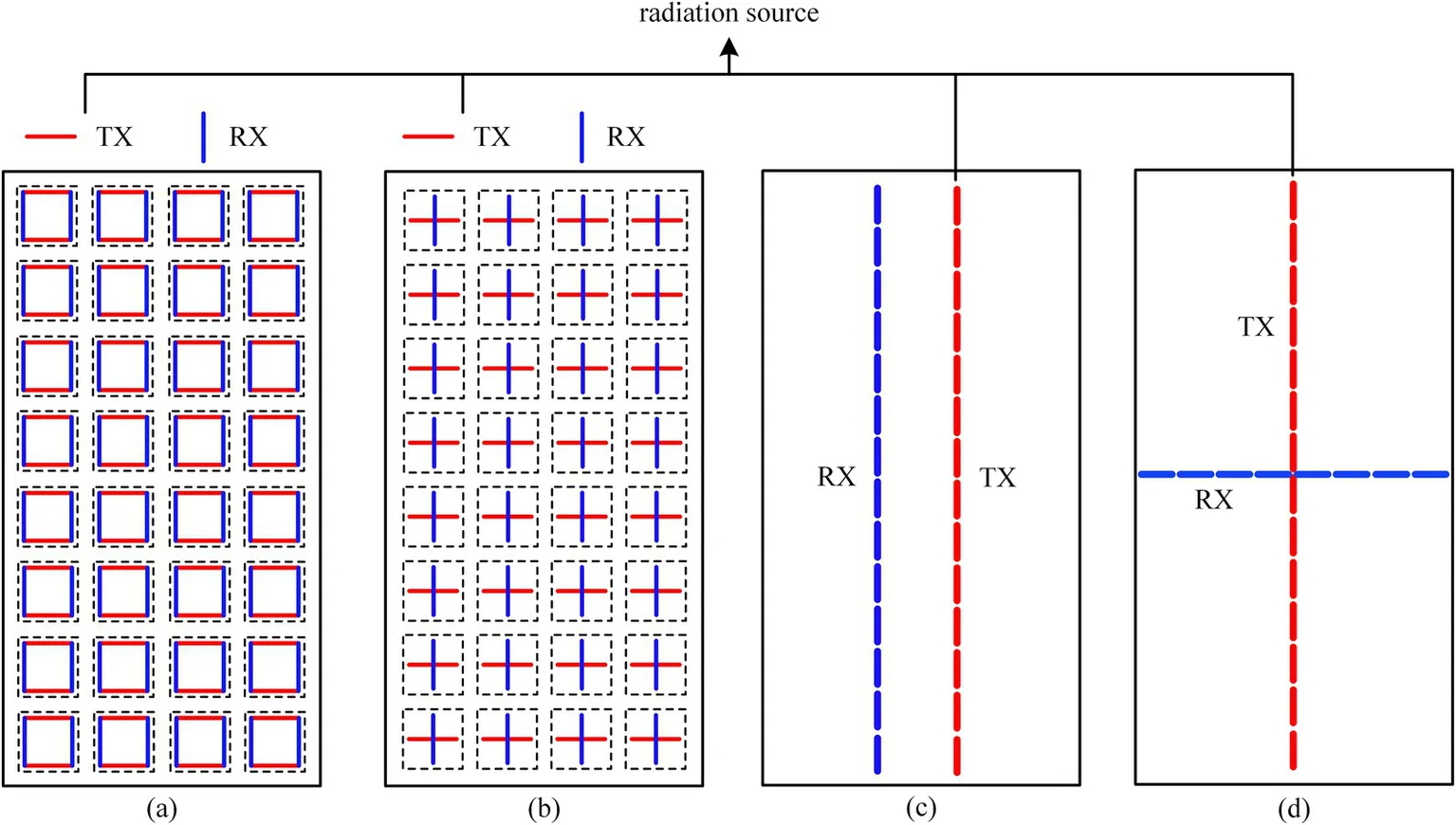
Representative antenna-array architectures of mainstream MMW systems: **(a,b)** planar TX/RX array configurations; **(c)** parallel linear TX/RX arrangement; and **(d)** orthogonal linear TX/RX arrangement.

Millimeter waves can penetrate conventional clothing while being reflected and absorbed differently by human skin and concealed objects, enabling contactless body-surface imaging (119). Passive systems detect naturally emitted and reflected MMW radiation, whereas active systems transmit low-power signals and reconstruct images from echoes reflected by the body surface and concealed objects.

### 5.5 Comparison of electromagnetic emission sources and technical characteristics of electromagnetic security gates

The electromagnetic emission sources, operating-frequency ranges, structural locations, and representative application scenarios of the principal categories of electromagnetic security gates are summarized in Table 4.

**Table 4:** Comparison of electromagnetic emission sources and exposure-relevant characteristics of major electromagnetic security-gate systems.

Device type	Frequency range	Principal electromagnetic emission source	Location of emission source	Representative application scenarios
WTMD	1–10 kHz	Transmitter coil	Embedded within one or both gate panels	Airports, railway stations, sports venues, and similar public-security settings
EM EAS	20 Hz–18 kHz	Transmitting antenna	Embedded within one or both gate panels	Libraries, small retail stores, convenience stores, and similar settings
AM EAS	58–132 kHz	Transmitting antenna	Embedded within one or both gate panels	Hospitals, large shopping centers, luxury-goods stores, and similar settings
RF EAS	1.8–10 MHz	Transmitting antenna	Embedded within one or both gate panels	Libraries, pharmacies, postal outlets, and similar settings
MW EAS	902–928 MHz and 2,400–2,500 MHz	Transmitting antenna	Embedded within one or both gate panels	Supermarkets, hypermarkets, warehouse-style retail stores, and similar settings
RFID	LF: 30–300 kHz HF: 3–30 MHz UHF: 860–960 MHz MW: 2.45 GHz	LF/HF: transmitting coil UHF/MW: dipole or patch-array antenna	Embedded within one or both gate panels	Identity verification, access control, and intelligent management systems
MMW systems	30–300 GHz	Active systems: transmitting antenna array; passive systems: no active emission source	Integrated into the vertical gate uprights and overhead section	Airports, border checkpoints, government buildings, and other high-security facilities

## 6 Methodologies for assessing electromagnetic exposure safety in electromagnetic security gates

### 6.1 Experimental measurement methods

Experimental methods quantify exposure-related physical parameters using standardized procedures, calibrated instruments, and predefined sampling strategies (120).

Forty-five-point torso and thirty-six-point head measurement schemes. IEC 62369-1 and EN 62369-1 specify grid-based procedures comprising 45 torso points and 36 head points (73, 121). These schemes characterize spatially averaged exposure under non-uniform fields. The torso grid covers the principal anatomical regions exposed near transmitting antennas, whereas the head grid represents regions associated with the central nervous system (CNS). The sampling locations are shown in Figures 10, 11.

**Figure 10:**
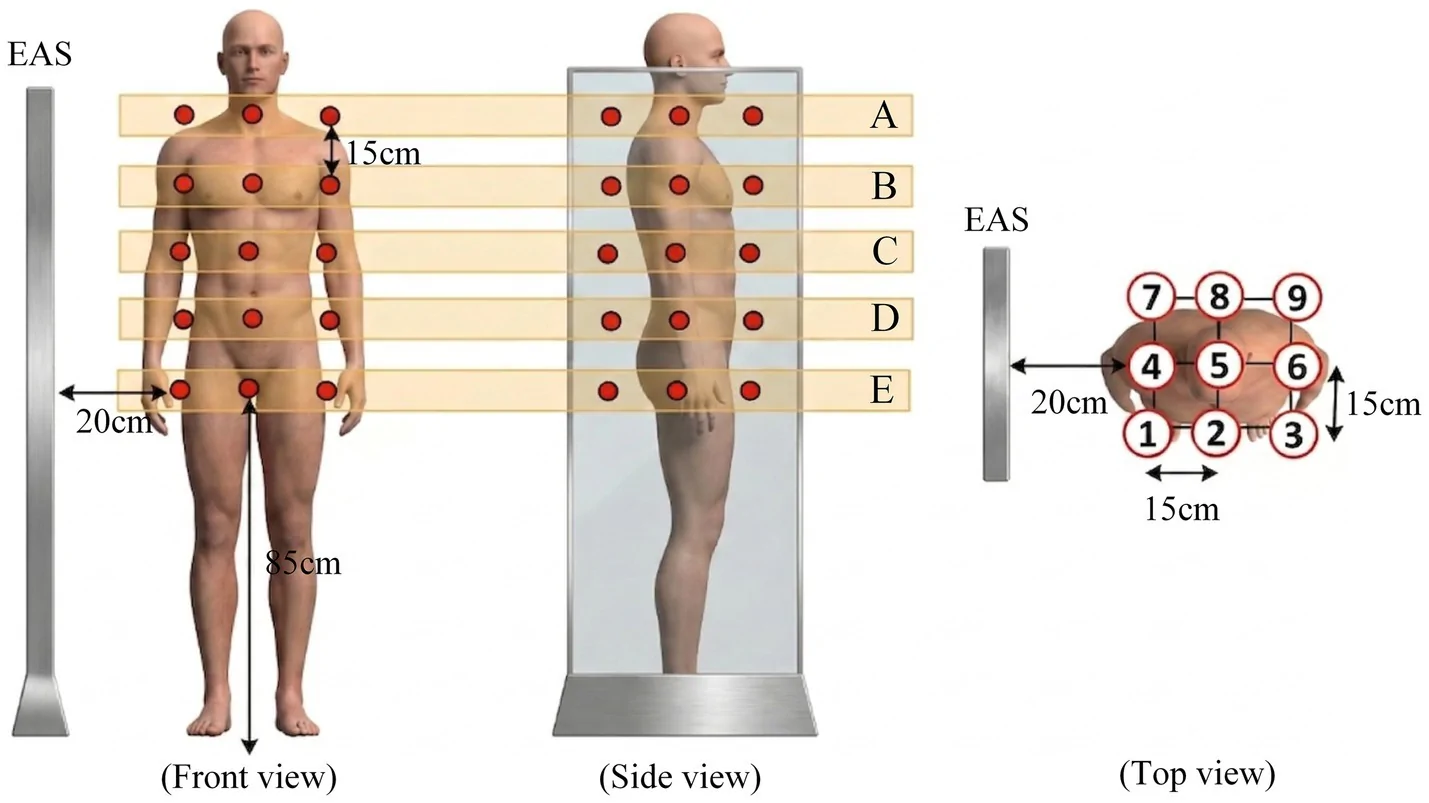
Forty-five-point measurement grid for the torso.

**Figure 11:**
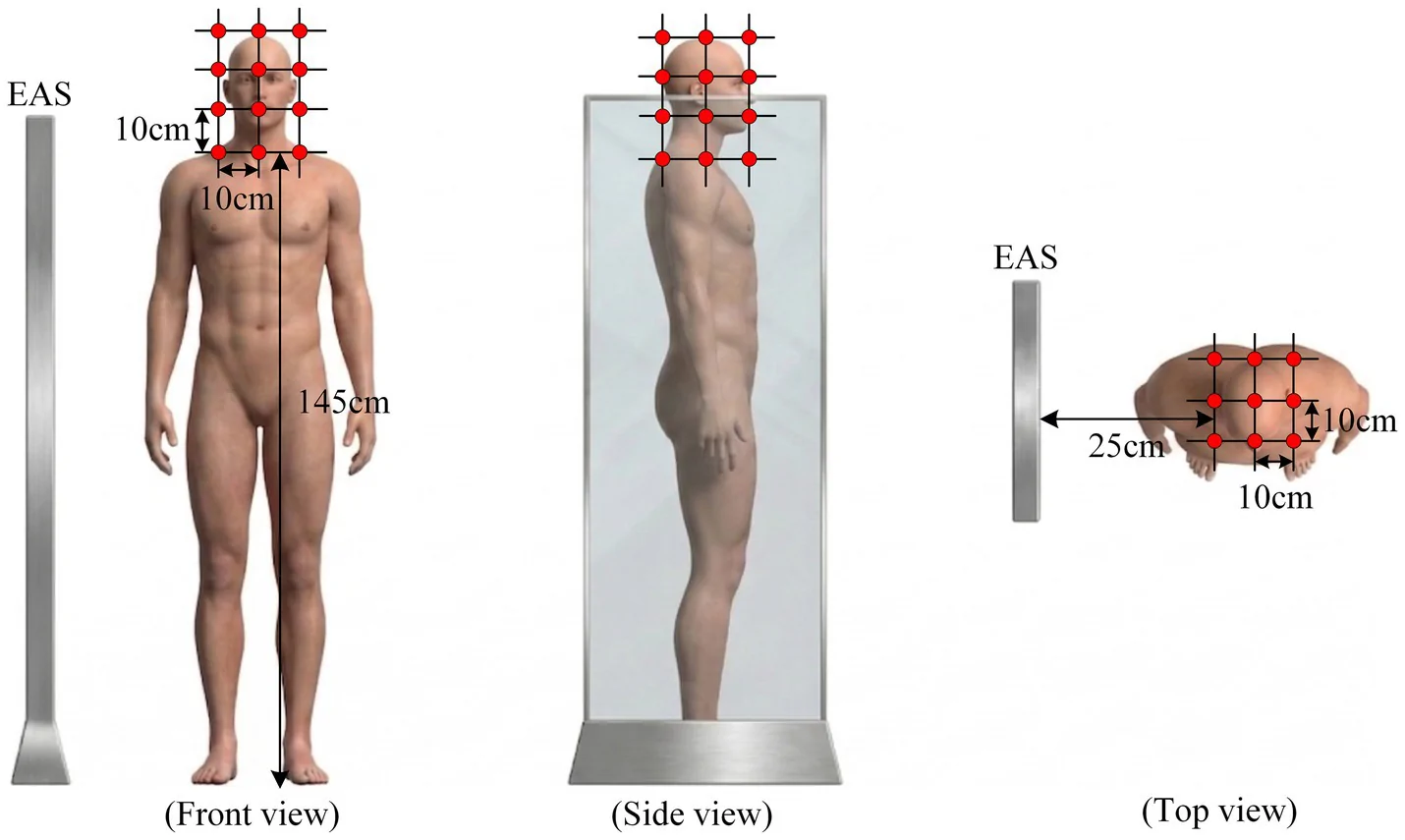
Thirty-six-point measurement grid for the head.

Simplified three-point measurement scheme. A simplified method measures the magnetic field at heights of 85, 115, and 145 cm, each 30 cm horizontally from the antenna center (108). The arithmetic mean of these measurements serves as a surrogate for the 45-point torso-grid result, reducing measurement time and cost. The sampling locations are shown in Figure 12.

**Figure 12:**
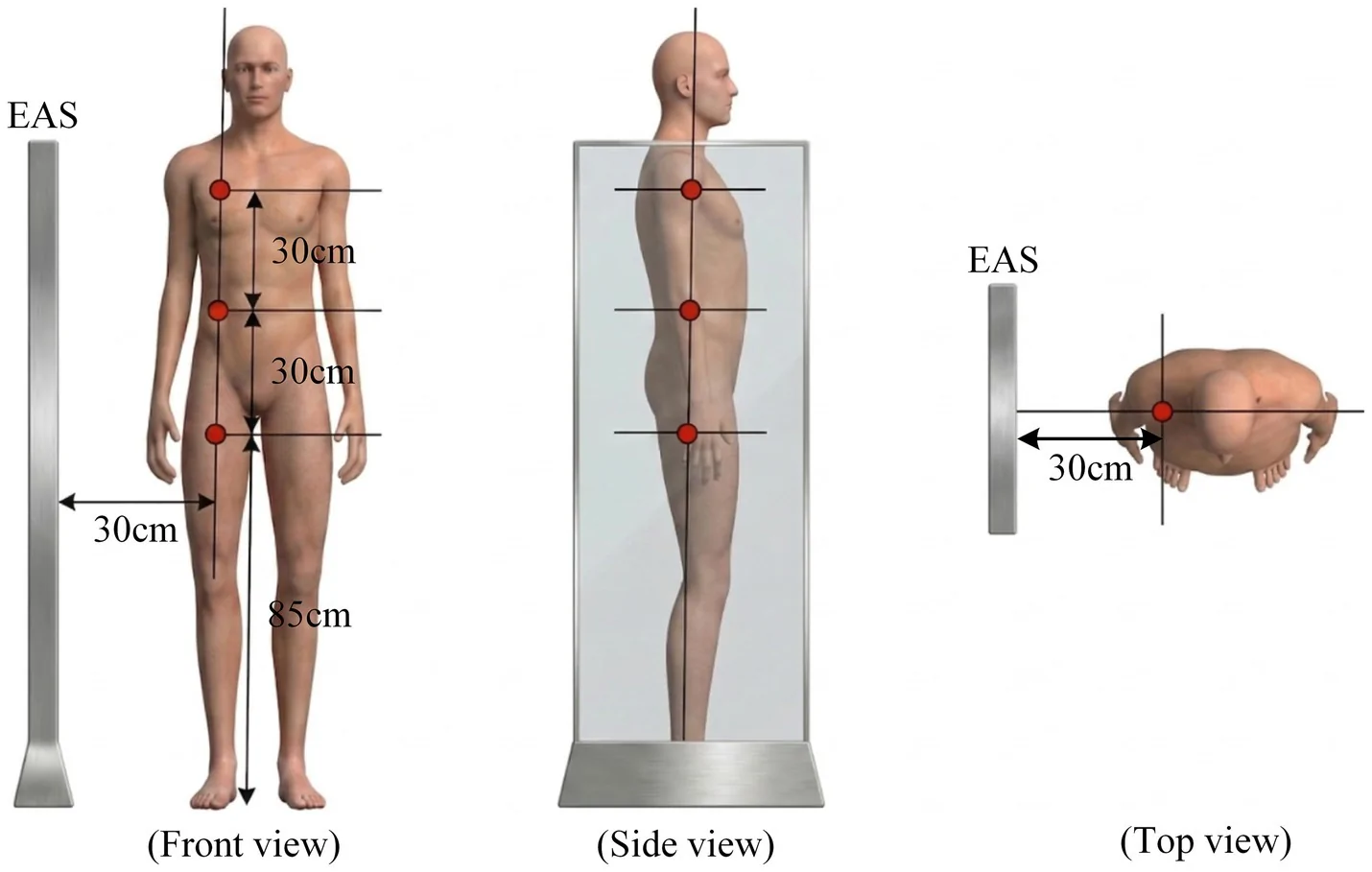
Sampling locations used in the simplified three-point measurement scheme.

Near-field scanning. For higher-frequency RFID, microwave, and MMW systems, two- or three-dimensional near-field scans can map incident or absorbed power-density distributions. Incident power density can be measured with appropriate probes, whereas absorbed power density is estimated using the electromagnetic properties of superficial tissues. For low-frequency WTMD and EAS systems, magnetic flux density, magnetic-field strength, or electric-field strength are generally more relevant metrics (122–124).

Bench and *in vivo* experiments. Bench tests use tissue-equivalent materials, anatomical phantoms, medical devices, and exposure simulators to evaluate biological responses or device interactions under controlled conditions (125). They provide reproducible assessment of EMI and device malfunction. *In vivo* studies provide more direct evidence of physiological or device-related responses under realistic exposure conditions. ISO 14117:2019 (126) and EN 50527 (127) specify electromagnetic-compatibility testing and risk-assessment procedures for individuals with implantable medical devices from product-compliance and occupational-safety perspectives, respectively.

### 6.2 Numerical simulation methods

Numerical simulation enables quantitative assessment of security-gate source fields and electromagnetic exposure within anatomical models. Depending on the method, simulations can characterize external fields, internal electric fields and current densities, and specific absorption rate (SAR) (128).

#### 6.2.1 Impedance method

This method represents the human body as a three-dimensional impedance network. Solving nodal voltages and branch currents yields internal electric-field and current-density distributions (129–132). It is particularly suitable for low- and intermediate-frequency exposure below approximately 10 MHz and can efficiently accommodate heterogeneous anatomical models.

#### 6.2.2 Finite-difference time-domain method (FDTD)

FDTD discretizes space and time and solves Maxwell’s curl equations iteratively (133, 134). It can accommodate complex anatomical structures and heterogeneous tissues and is particularly useful for time-dependent phenomena, including transient responses to pulsed fields.

#### 6.2.3 Scalar-potential finite-difference method (SPFD)

SPFD uses the quasi-static characteristics of low-frequency fields to reformulate the exposure problem in terms of scalar potential (135). It reduces computational complexity and is well suited to estimating internal electric fields and induced current densities at low frequencies.

#### 6.2.4 Finite-element method (FEM)

FEM discretizes the computational domain into finite elements and is well suited to complex geometries and irregular tissue interfaces (3). It also facilitates multiphysics coupling between electromagnetic fields and heat transfer. Boundary conditions must be selected according to the frequency range and exposure configuration.

The choice of numerical method depends primarily on operating frequency, geometric complexity, and exposure configuration. Other approaches, such as the three-dimensional indirect boundary integral equation method (3D-IBIEM), may also be used for specific source representations and computational requirements (136).

### 6.3 Electromagnetic exposure simulators

Exposure simulators reproduce representative electromagnetic environments generated by security gates through hardware or numerical approaches (131, 136–138). Hardware simulators typically use a waveform generator, power amplifier, and coil system, as illustrated in Figure 13, to reproduce representative waveforms, field strengths, and spatial distributions. Numerical simulators use methods such as 3D-IBIEM or FDTD and permit systematic variation of frequency, field strength, and source configuration. These platforms support reproducible testing of EMI, medical-device safety, and exposure scenarios that are difficult to reproduce using commercial security gates.

**Figure 13:**
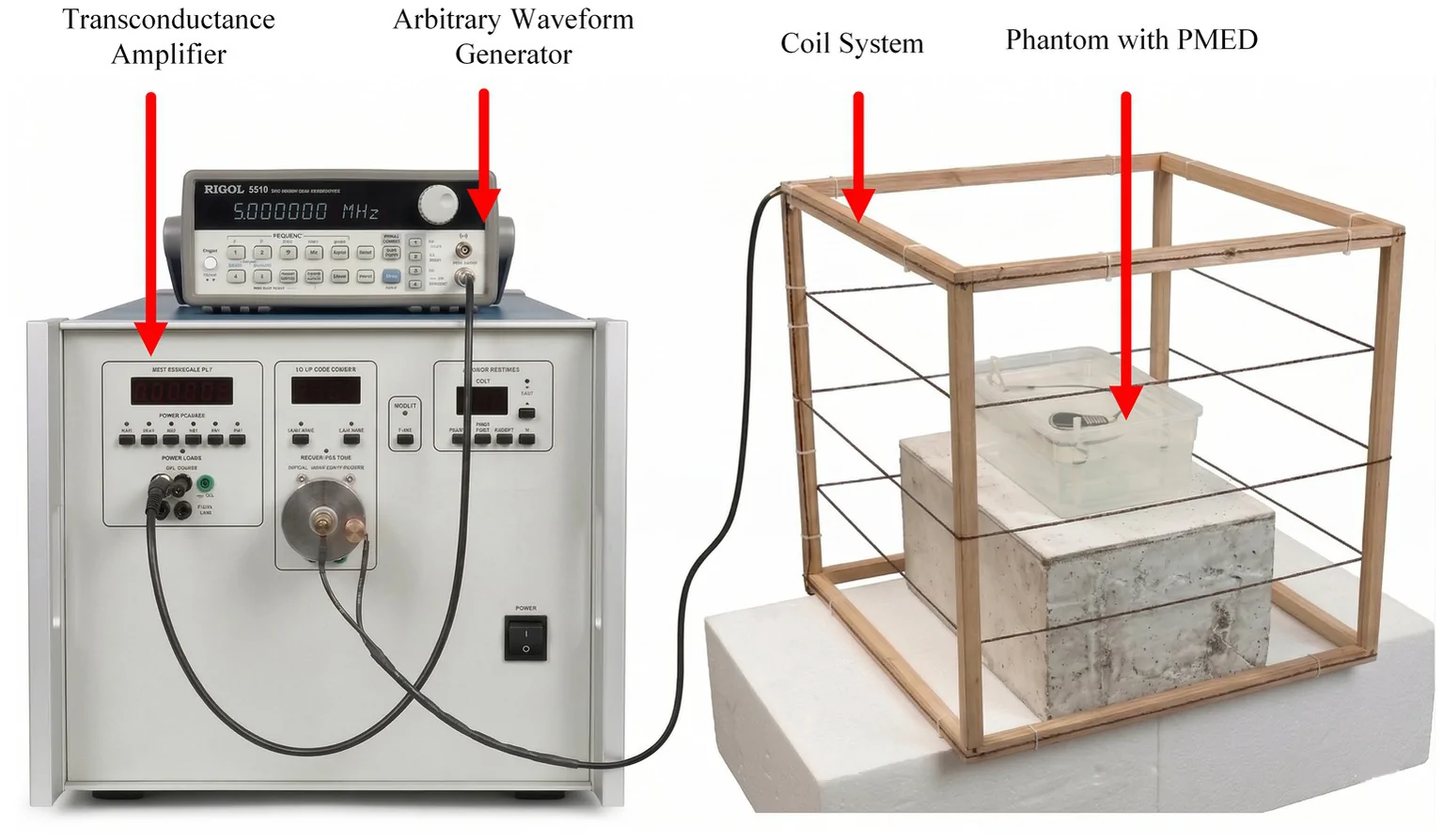
Example of a test setup for an electromagnetic security-gate exposure simulator.

## 7 Experimental measurement results for the electromagnetic exposure safety assessment of electromagnetic security gates

### 7.1 Electromagnetic-field characteristics and spatial distributions

The regions surrounding the operating zones of most electromagnetic security gates lie within the reactive near-field regime, whereas some higher-frequency devices may involve radiating near-field exposure (120, 121, 139).

#### 7.1.1 WTMDs

The magnetic fields generated by WTMDs commonly feature continuous-wave sinusoidal signals, pulse-modulated waveforms with specified duty cycles, and sawtooth waves containing high-frequency harmonic components. Multi-zone detection systems may additionally exhibit phase differences among individual detection zones. Peak magnetic-field strengths are concentrated in the vicinity of the transmitting antennas or gate panels and decrease rapidly with increasing distance from the panels. The field strength at the center of the passageway is typically less than one-tenth of the peak value measured near the gate panels. Along the vertical axis, the magnetic field remains relatively stable within the torso-height range of approximately 100–160 cm. In systems equipped with two transmitting panels, a local minimum in field strength may occur at the center of the passageway, while the individual magnetic-field components exhibit approximately symmetric spatial distributions (1, 140), as illustrated in Figure 14.

**Figure 14:**
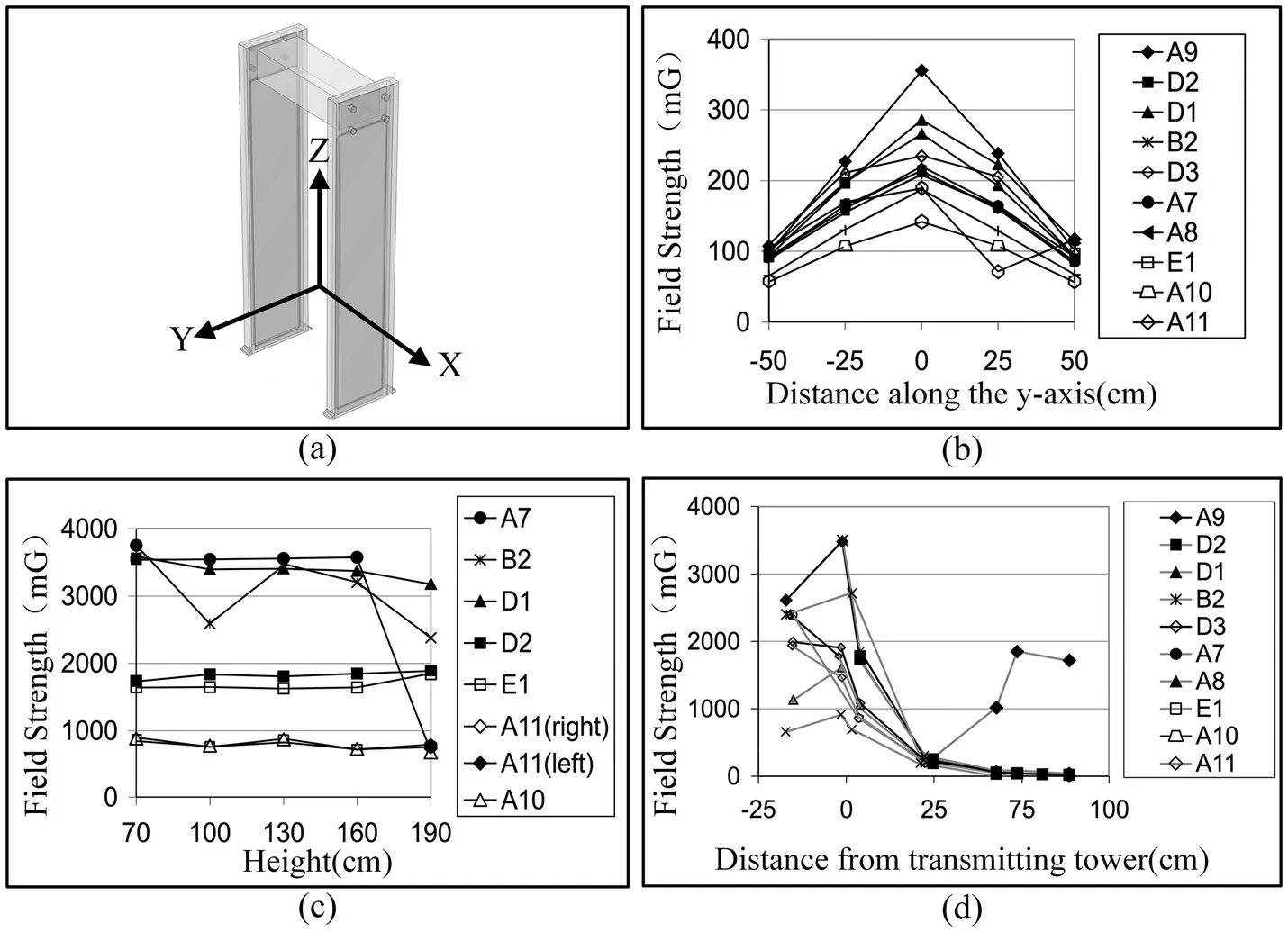
Spatial distribution of the magnetic field generated by a WTMD [Source: Ref. (1)]: **(a)** coordinate system of the WTMD; **(b)** magnetic-field strength along the *y*-axis at *x* = 0 and *z* = 130 cm; **(c)** relationship between magnetic-field strength and height at *x* = 0 and *y* = 0; and **(d)** magnetic-field strength along the *x*-axis at a height of 130 cm.

#### 7.1.2 EAS systems

EAS excitation waveforms vary by technology: EM and MW systems generally use continuous-wave excitation, AM systems employ pulse-modulated waveforms, and RF systems use frequency-swept signals. The magnetic fields generated by EAS systems may exhibit an elliptically rotating pattern. Peak field strengths are concentrated in the vicinity of the transmitting antennas or gate panels. The field magnitude decreases sharply within the immediate near-field region and more gradually at greater distances, producing a spatial gradient centered on the transmitting panel. Accordingly, field strengths at the center of the passageway are substantially lower than those measured in the near-field region adjacent to the gate panels (141, 142).

Reported measurements indicate that the magnetic-field magnitudes and attenuation characteristics of the different EAS technologies generally follow the order EM > AM > RF > MW under the corresponding measurement conditions (143–145), as illustrated in Figure 15. This comparison should be interpreted with caution because magnetic-field strength, electric-field strength, and power density represent distinct exposure metrics and are not directly interchangeable across different frequency ranges. Using the ICNIRP exposure guidelines as reference criteria, minimum compliance distances of 111, 77, and 35 cm were proposed for EM, AM, and RF systems, respectively (71, 146). Based on detailed measurements of the spatial fields generated by EAS systems, a quantitative relationship was derived for distances greater than 50 cm from the nearest gatepost (53). In this region, the magnetic flux density was found to decrease approximately in proportion to (*r*^-2.9^), where *r* denotes the distance from the measurement point to the nearest gatepost. This empirical dependence is broadly consistent with the inverse-cube distance dependence (*r*^−3^) characteristic of an equivalent magnetic-dipole model (53, 139). The small difference from the ideal *r*^−3^ dependence may reflect the extended two-post geometry of practical EAS systems and the empirical nature of the fit, which was derived from a limited number of gate models (53).

**Figure 15:**
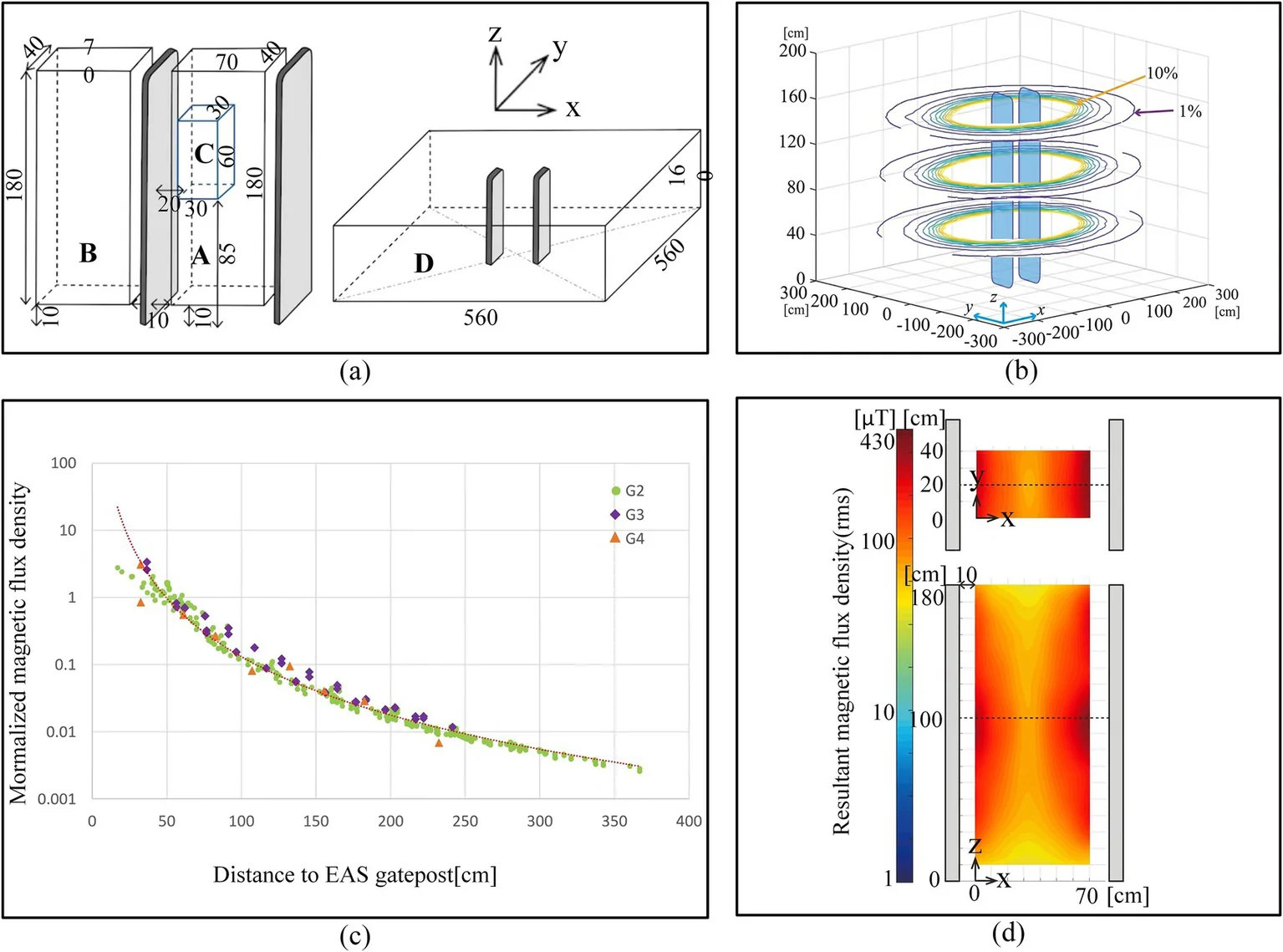
Spatial distributions of the fields generated by EAS systems: **(a)** Schematic representation of the measurement region; **(b)** Contour map of magnetic flux density surrounding the EAS system in region D; **(c)** Relationship between magnetic flux density and the distance to the nearest gate pillar; and **(d)** Magnetic-field distribution of the EAS system in region A, expressed as the root-mean-square resultant magnetic flux density. The xy-plane section is evaluated at z = 100 cm, and the xz-plane section is evaluated at y = 20 cm. Panels **(a–d)** are reproduced from Figures 1, 5, 6, and 3, respectively, of Ikuyo et al. (53), distributed under the Creative Commons Attribution 4.0 International License (CC BY 4.0).

#### 7.1.3 RFID systems

The electromagnetic fields generated by RFID systems are distributed radially around the transmitting antennas. Field strengths are highest directly in front of the antennas and progressively decrease with increasing distance within the passageway. Among the RFID systems considered, lower-frequency devices exhibit comparatively uniform magnetic-field distributions, whereas higher-frequency devices produce more directional electric-field distributions (109, 111).

### 7.2 Experimental evidence of electromagnetic interactions between electromagnetic security gates and medical devices

Medical devices relevant to security-gate exposure include active implantable medical devices (AIMDs), such as pacemakers (PMs), implantable cardioverter-defibrillators (ICDs), neurostimulators, and implanted infusion pumps; body-worn devices such as insulin pumps; and passive metallic implants such as joint prostheses and spinal implants. Figure 16 shows representative device types and anatomical locations. FDA/CDRH reports and the clinical and laboratory literature indicate that electromagnetic fields from some security gates can affect certain medical devices (147–150).

**Figure 16:**
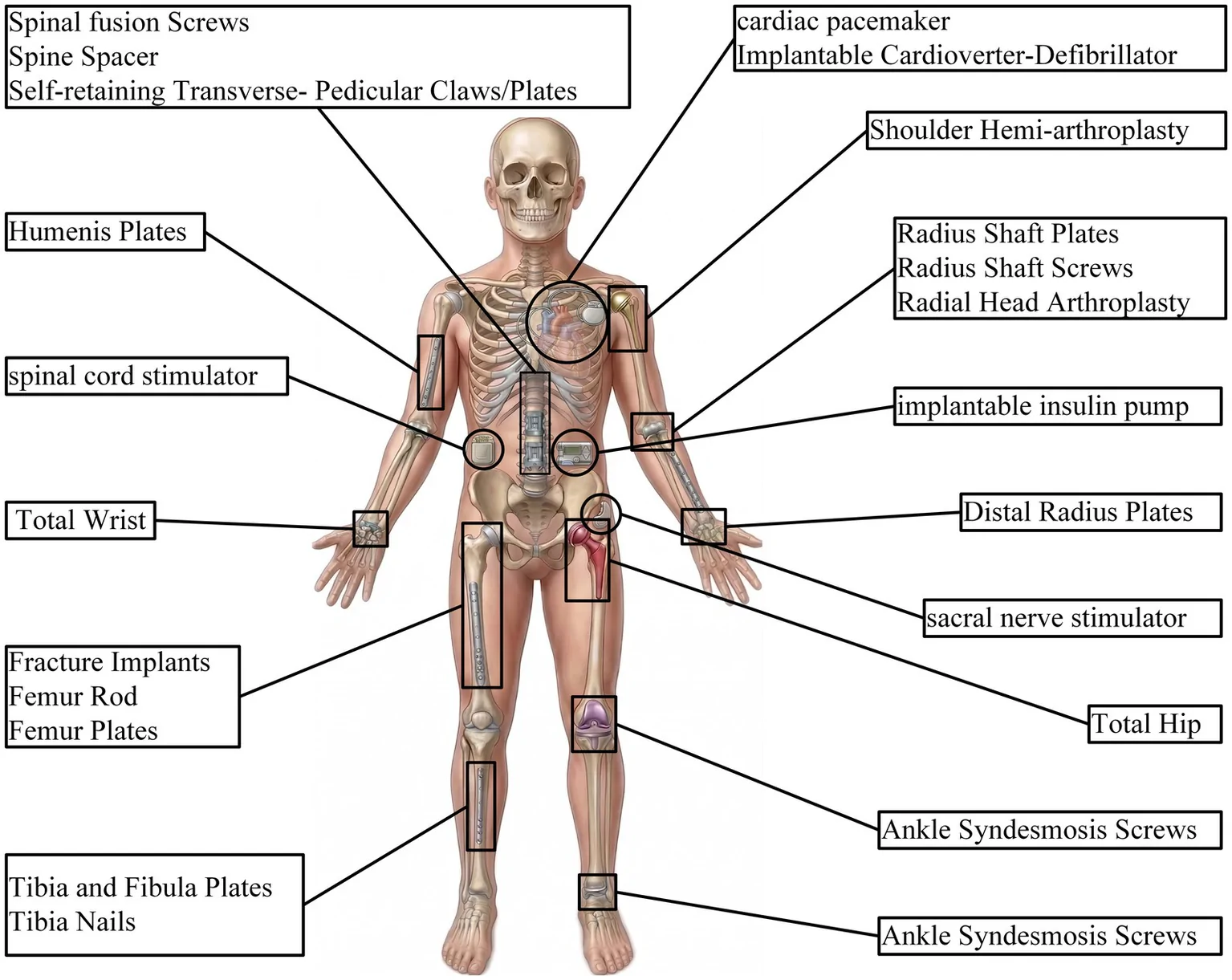
Typical locations of medical devices and passive implants relevant to the assessment of interactions with electromagnetic security gates.

For AIMDs and body-worn medical electronic devices, the principal endpoint is functional electromagnetic interference (EMI). The likelihood of EMI depends on the operating frequency of the field source, signal-modulation waveform, field strength and spatial distribution, exposure distance, operating parameters of the security gate, device type, and device operating mode (11). For passive metallic orthopedic implants, the primary consideration is not functional interference but the influence of the implant on WTMD detection signals and alarm responses.

#### 7.2.1 WTMDs

Under the standard operating conditions examined in previous studies, WTMDs generally exhibited satisfactory electromagnetic compatibility with PMs and ICDs. Adverse events such as pacing inhibition, abnormal sensing, and spontaneous reprogramming of device parameters were not observed (84, 85). However, exposure-simulator studies have shown that transient EMI may still occur in a small number of PMs and implantable neurostimulators under specific waveform, field-strength, and exposure conditions. For passive metallic orthopedic implants, WTMD alarm activation depends on implant mass, volume, geometry, material properties, and magnetic permeability, as well as on the preset sensitivity of the WTMD and the speed at which the individual passes through the gate (86–88).

#### 7.2.2 EAS systems

Under the tested conditions, MW and RF EAS systems exhibited a comparatively low probability of inducing functional EMI in AIMDs. By contrast, EM and AM EAS systems, particularly those employing high-energy pulsed or amplitude-modulated waveforms, may induce relatively high peak voltages in implanted lead loops (89–91). Clinical manifestations reported in previous case studies and experimental investigations include (1) ICD oversensing followed by inappropriate shock delivery; (2) inhibition of PM output, resulting in palpitations, dyspnea, dizziness, or, in severe cases, syncope (92); and (3) unintended activation of spinal-cord stimulators accompanied by neurological symptoms (93). These risks are closely associated with close-range dwell time, implanted-lead configuration, and device-sensing mode, and should not be directly extrapolated to routine passage through EAS systems.

#### 7.2.3 RFID systems

PMs and ICDs appear to be most susceptible to modulated LF RFID signals, followed by HF RFID signals. Under the device configurations and test conditions examined in previous studies, no clear interference effects were identified for UHF RFID systems or RFID devices operating in continuous-wave mode (94). Most implantable neurostimulators exhibited satisfactory electromagnetic compatibility with the RFID systems tested, although weak interference was observed in individual devices at specific frequencies, such as 134 kHz (95).

Taken together, the studies summarized in Table 5 indicate that interference risk depends primarily on operating frequency, signal-modulation strategy, antenna topology, and exposure duration. Low-frequency pulsed or amplitude-modulated EAS signals and prolonged close-range exposure warrant particular attention because they may increase the voltage induced in implanted leads. In addition, rectangular or approximately rectangular loop antennas may generate steep magnetic-flux-density gradients near the edges of gate panels when driven by alternating currents (96).

**Table 5:** Progress in studies of interactions between medical devices and electromagnetic security gates.

Security-gate type	Medical device or implant type	Study design	Assessment protocol	Observed outcome	Principal conclusion	References
WTMD	PM	*In vivo* study involving 103 patients	Bidirectional passage through the gate with telemetry monitoring	No pacing inhibition or device reprogramming was observed	WTMD exposure did not adversely affect PM function under the tested conditions	(84)
EAS (EM/AM/RF)	PM	In vivo study involving 32 patients; bench testing of 28 devices	In vivo: routine passage with telemetry monitoring; bench testing: devices moved near the EAS system at a rate corresponding to cardiac activity	No significant interference from AM or RF systems; interference was observed for EM systems	EM systems may pose a greater risk to patients with dual-chamber PMs; unipolar PMs appear more susceptible to inhibition	(97)
EAS (EM/AM/RF/MW)	PM	Bench testing of five devices	PMs connected to a simulator and moved through the center of the gate, along multiple axes, and adjacent to the antenna	MW and RF systems produced minimal effects, whereas AM and EM systems caused more frequent interference	EAS systems may induce EMI at very short distances	(98)
EAS	ICD	Clinical case report	Retrospective analysis of a severe adverse event following accidental exposure	Inappropriate ICD discharge attributable to EMI	EAS exposure may cause ICD oversensing	(92)
WTMD	Orthopedic implant	In vivo study involving 16 patients; bench testing with implants attached to healthy volunteers	Alarm activation assessed at different WTMD sensitivity settings	Single implants triggered alarms only at high sensitivity settings; multiple implants were more readily detected	Alarm activation depends on the number of implants and the sensitivity setting of the WTMD	(167)
WTMD	Orthopedic implant	In vivo study involving 15 patients; bench testing with implants attached to healthy volunteers	Patients and volunteers carrying prostheses of different masses passed repeatedly through the WTMD	All metallic prostheses weighing more than 195 g triggered an alarm	Orthopedic implants exceeding a critical mass are likely to be detected by standard WTMDs	(168)
EAS (RF)	Spinal-cord stimulator	Clinical case report	Retrospective analysis of a severe adverse event following accidental exposure	The patient experienced an abrupt electrical sensation, loss of consciousness, and neurological symptoms while passing through the EAS system	RF EAS exposure may trigger unintended activation of a spinal-cord stimulator	(93)
EAS (EM/AM/RF)	PM/ICD	In vivo study involving 50 p.m. users and 25 ICD users	Participants walked through, stood within, and rotated inside EAS systems under telemetry monitoring	No effects were observed in ICDs; PM interference was observed in 48, 2, and 0 patients exposed to EM, AM, and RF systems, respectively	High-energy pulsed low-frequency EAS systems may interfere with a substantial proportion of PMs	(89)
EAS (AM)	PM	Clinical case report	Retrospective analysis of a severe adverse event following accidental exposure	The patient experienced symptoms while passing through an AM system	AM EAS systems may interfere with PM function	(169)
EAS (EM/AM)	ICD	In vivo study involving 170 patients	Participants walked slowly through the center of the gate for 10–15 s and remained within 6 inches of the antenna for 2 min	Normal function was maintained during slow passage; prolonged close-range exposure resulted in interference in 19 patients	Remaining close to an EAS antenna may result in inappropriate shocks or inhibition	(90)
EAS (EM/AM)	PM	In vivo study involving 204 patients	Participants remained within the EAS field for up to 30 s; different sensing modes were compared	Overall interference rate: 17%	EM systems produced approximately twice as much interference as AM systems; unipolar PMs were approximately twice as susceptible as bipolar PMs; dual-chamber PMs were more susceptible	(99)
WTMD	Orthopedic implant	In vivo study involving 12 patients; bench testing in which healthy volunteers carried 60 implants	Relationships between detection rate and implant mass, composition, and passage speed were analyzed	Detection was inconsistent; some implants triggered alarms	Larger and more readily magnetizable implants are more likely to be detected; implant mass and passage speed influence detection	(86)
WTMD	PM/ICD	In vivo study involving 200 p.m. users and 148 ICD users	Participants remained within the WTMD for at least 20 s and rotated through 360°	No inhibition, abnormal sensing, parameter changes, or device reprogramming were observed	Standard airport WTMDs did not produce detectable EMI in PMs or ICDs under the tested conditions	(85)
WTMD	Orthopedic implant	In vivo study involving 129 patients	Participants passed through a high-sensitivity WTMD	More than half of the orthopedic implants were detected	Total joint prostheses were frequently detected; lower-limb implants were more readily detected than upper-limb or spinal implants	(170)
EAS	PM/ICD	Clinical case reports	Retrospective analysis of adverse events following accidental exposure	One PM user lost consciousness and collapsed; one ICD user received two shocks	EAS systems may adversely interact with cardiac implantable electronic devices	(91)
WTMD	Orthopedic implant	Epidemiological survey	Mailed questionnaire survey of 200 patients more than one year after surgery, selected from a hospital database	Forty-seven percent of implants were detected; 72% of patients reported no substantial inconvenience	Orthopedic implants may be detected relatively frequently, but travel disruption is generally limited	(171)
WTMD	Implantable neurostimulator	Exposure-simulator study	Three neurostimulators were exposed to 12 WTMD simulator configurations	One neurostimulator exhibited possible EMI	Device modes and parameter settings should be evaluated before deployment of the security system	(138)
RFID	PM/ICD	Exposure-simulator study	Fifteen PM models and 15 ICD models were exposed to 13 RFID simulator configurations	PMs and ICDs were most susceptible to LF RFID, followed by HF RFID; no response was observed for UHF or continuous-wave RFID	UHF and continuous-wave RFID systems may be preferable where AIMD compatibility is a priority	(94)
RFID	Implantable neurostimulator	Exposure-simulator study	Six neurostimulators were exposed to 22 RFID simulator configurations	Interference was observed in only one device at 134 kHz	Most implantable neurostimulators exhibit satisfactory compatibility with RFID systems	(95)
EAS	ICD	Clinical case report	Retrospective analysis of an adverse event following prolonged proximity to an EAS system	The patient remained near the EAS system and experienced an ICD shock	EAS exposure may interfere with ICD function	(172)
WTMD	Spinal implant	In vivo study involving 40 patients; bench testing in which healthy volunteers carried 60 implants	Alarm activation assessed at different WTMD sensitivity settings	No spinal implants were detected	WTMDs may fail to detect modern spinal and foot-and-ankle implants	(173)
EAS	PM/ICD	In vivo study involving 11 p.m. users and 13 ICD users	Routine passage and a 1-min dwell period with telemetry monitoring	Interference occurred in one unipolar PM	Unipolar PMs appear more susceptible than bipolar PMs and ICDs	(100)
WTMD	Metallic prosthetic implant	Bench testing	Multiple prosthetic devices were passed sequentially through the WTMD	None of the tested prosthetic devices were detected	Some WTMD configurations may fail to detect metallic prosthetic implants	(88)
WTMD	Foot-and-ankle implant	In vivo study involving 153 patients; bench testing	Detection rates were compared between isolated hardware and implants covered with muscle-equivalent tissue	No alarms were triggered during patient passage; detection rates were higher in bench testing	The capacitive and conductive properties of biological tissues may attenuate WTMD detection signals	(174)
WTMD	PM, ICD, implantable neurostimulator, and insulin pump	Exposure-simulator study	Medical devices were exposed to magnetic fields generated by a WTMD simulator	Transient interference was observed in only two PMs and one neurostimulator	Maintaining an appropriate distance and minimizing exposure duration may reduce risk	(4)

Study-level characteristics are presented to facilitate comparison of study design and exposure conditions. Evidence strength was appraised at the body-of-evidence level rather than assigned to individual studies; domain-level ratings are summarized in Section 7.3 and Table 1.

### 7.3 Critical appraisal of experimental evidence

Within the framework described in Section 2.3, experimental evidence was evaluated in two domains: direct characterization of security-gate electromagnetic fields and experimental assessment of medical-device interactions.

Field measurements of WTMD and EAS systems consistently show the highest exposure near transmitting antennas or gate panels, with marked attenuation toward the passageway center (1, 53, 140–146). Quantitative comparison is nevertheless limited by differences in frequency, waveform, antenna configuration, measurement position, and exposure metric. Magnetic flux density, magnetic-field strength, electric-field strength, and power density are also not directly comparable across frequency ranges. Experimental characterization remains less extensive for MMW screening than for WTMD, EAS, and RFID systems. This evidence domain was therefore rated as moderate in strength: the principal spatial patterns are consistent and directly relevant, but cross-study comparability and technology coverage remain limited.

Evidence on medical-device interactions includes *in vivo* studies, bench tests, simulator experiments, and clinical case reports. Relatively large WTMD studies generally found no clinically relevant interference with PMs or ICDs under the tested conditions (84, 85). EAS studies, however, reported greater interference under selected conditions, particularly with low-frequency EM or AM systems, unipolar or dual-chamber pacing configurations, prolonged close-range exposure, and pulsed or amplitude-modulated waveforms (89, 90, 97–100). RFID studies similarly indicate greater susceptibility at LF and, to a lesser extent, HF frequencies, with no clear responses under the tested UHF or continuous-wave conditions (94, 95). These differences are better explained by variations in technology, waveform, field strength, distance, duration, device configuration, and test protocol than by conflicting evidence.

Clinical case reports confirm that severe interference can occur under particular circumstances but cannot establish event incidence or routine-passage risk. Passive metallic implants should be interpreted separately because the available studies primarily address WTMD alarm activation rather than functional EMI.

Overall, experimental evidence on interactions between security gates and active medical devices was rated as moderate. Its strengths include directly relevant *in vivo* studies, complementary bench and simulator data, and consistent identification of frequency, waveform, distance, dwell time, and device configuration as major determinants. A high-strength rating was not assigned because protocols remain heterogeneous, device generations and operating modes vary, some adverse-event evidence relies on case reports, and independent replication is limited. The evidence therefore supports a conditional interpretation: routine passage generally presents lower interference potential than prolonged close-range exposure, whereas specific low-frequency or modulated configurations warrant greater caution.

## 8 Numerical simulation results for the electromagnetic exposure safety assessment of electromagnetic security gates

### 8.1 Equivalent radiation sources and human-body-equivalent antennas

Security-gate sources can be represented as electrically small or electrically large antennas. Electrically small sources, whose dimensions are substantially smaller than the operating wavelength, can often be approximated as magnetic dipoles with magnetic-field-dominated reactive near fields (111). EM, AM, and RF EAS systems and LF/HF RFID systems are therefore commonly modeled as rectangular or approximately rectangular loop antennas (112).

Electrically large antennas are more relevant to shorter-wavelength systems such as UHF RFID and MW EAS, where radiative coupling and directionality become more important. For WTMDs with complex crossed-coil structures, measured field distributions have been combined with the Biot–Savart law and least-squares fitting to derive equivalent current sources for numerical modeling (140, 151).

Conventional human-body-equivalent antennas designed for far-field plane-wave exposure are not directly applicable to security-gate near fields. A model comprising two orthogonal miniature circular loops with an outer radius of 5 cm was therefore proposed for near-field exposure (139). It reproduces approximately *r*^−3^ magnetic-field and *r*^−2^ electric-field attenuation, and the resulting induced-current measurements agreed closely with those obtained in human subjects at comparable distances.

### 8.2 Human electromagnetic dosimetry: numerical simulations and results

Table 6 summarizes numerical dosimetry studies separately for adult/general-population models (Table 6A) and susceptible, implant-bearing, or mixed-population models (Table 6B).

**Table 6:** Summary of numerical dosimetry studies of human exposure to electromagnetic security gates.

Security-gate type	Frequency	Human model	Numerical method	Applicable metric/limit	Principal dosimetric result (ER)	References
(A) Adult/general-population models						
WTMD	60 Hz	Adult model	Impedance method	No matched limit reported	*J* = 3.5 × 10^−4^ A/m^2^; ER = N/A	(140)
RFID	13.56 MHz	Simplified adult model	NR	SAR_WB_: GP 0.08 W/kg; OE 0.4 W/kg	SAR_WB_ = 2.4 × 10^−5^ W/kg; ER = 0.0003 × (GP)/0.00006 × (OE)	(112)
EAS (EM)	366 Hz	TARO adult model	Impedance method	Internal *E*: GP 0.4 V/m; OE 0.8 V/m	*E* = 0.017 V/m; ER = 0.043 × (GP)/0.021 × (OE)	(53)
EAS (EM)	14 kHz	TARO adult model	Impedance method	Internal *E*: GP 1.9 V/m; OE 3.8 V/m	*E* = 0.88 V/m; ER = 0.46 × (GP)/0.23 × (OE)	(53)
(B) Susceptible, implant-bearing, and mixed-population models						
EAS	30 kHz	Adult, 10-y, and 5-y models	Impedance method	Brain *J*_1cm_^2^: GP 60 mA/m^2^	Adult 17.63 (0.29×); 10-y 64.64 (1.08×); 5-y 98.93 mA/m^2^ (1.65×)	(154)
RFID	125 kHz	Adult model with metallic implant	FDTD	*J*_1cm_^2^: GP 250 mA/m^2^	*J*_1cm_^2^ = 3,300 mA/m^2^; ER = 13.2×	(159)
RFID	13.56 MHz	Adult model with metallic implant	FDTD	SAR₁₀g: GP head/torso 2 W/kg	SAR₁₀g = 7.31 W/kg; ER = 3.66×	(159)
WTMD	940 Hz	Pregnant-woman/fetal models	Impedance method	*B*: ICNIRP 6.25 μT; IEEE 730 μT; *J*: ICNIRP 2 mA/m^2^; E: IEEE 277 mV/m	*B*: 9.92/29.4 μT → 1.59×/0.040×; *J* = 3.09 mA/m^2^ → 1.55×; *E* = 16.61 mV/m → 0.060×	(151)
EAS (RF)	10 MHz	Adult/child models	FDTD/FEM	SAR_WB_ 0.08 W/kg; SAR₁₀g 2 W/kg; *J* 20 A/m^2^	1.46 × 10^−4^ W/kg (0.0018×); 5.53 × 10^−3^ W/kg (0.0028×); 1.16 A/m^2^ (0.058×)	(3)
EAS (AM)	125 kHz	Infant model	SPFD	No matched limit reported	*E*_99_CNS_ = 2 V/m; SAR₁₀g = 8.82 × 10^−4^ W/kg; *J*_max_ = 1.05 A/m^2^; ER = N/A	(152)
EAS (AM/RF)	125 kHz	Adult/infant models	SPFD	*E*_99_CNS/PNS_ = 32 V/m	*E*_99_CNS/PNS_ = 14.16 V/m; ER = 0.44×	(153)
EAS (AM/RF)	13.56 MHz	Adult/infant models	SPFD	SAR_WB_: 0.08 W/kg (GP); SAR₁₀g: 2 W/kg (GP, head/torso)	SAR_WB_ = 9.07 × 10^−4^ W/kg (0.011×); SAR₁₀g = 9.2 × 10^−3^ W/kg (0.0046×)	(153)
EAS (AM/RF)	125 kHz	Pregnant-woman/twin-fetus models	SPFD	*E* = 32 V/m	*E* = 0.45 V/m (0.014×)	(135)
EAS (AM/RF)	13.56 MHz	Pregnant-woman/twin-fetus models	SPFD	SAR_WB_: 0.08 W/kg (GP); SAR₁₀g: 2 W/kg (GP, head/torso)	SAR_WB_ = 0.006 W/kg (0.075×); SAR₁₀g = 0.088 W/kg (0.044×)	(135)
EAS (AM)	58 kHz	Adult/child models; worst-case posture	Numerical dosimetry	ICNIRP BR; EI = 1	Maximum EI = 22.5 × (GP)/4.5 × (OE)	(175)

ER, exposure ratio, calculated as the reported dosimetric value divided by the corresponding exposure restriction or reference level. ER is dimensionless and is reported as a fold value (×); ER > 1 indicates exceedance of the compared limit, whereas ER < 1 indicates a value below the limit. B, magnetic flux density; E, electric-field strength; J, induced current density; where an averaging basis was explicitly reported, it is indicated by the corresponding subscript; J_1cm_^2^, induced current density averaged over a 1-cm^2^ cross-section perpendicular to the direction of current flow; J_max_, maximum induced current density as reported in the original study; SAR_WB_, whole-body-averaged specific absorption rate; SAR_10g_, specific absorption rate averaged over 10 g of contiguous tissue; E_99_CNS_ and E_99_CNS/PNS_, 99th-percentile internal electric-field strength in the central nervous system and central/peripheral nervous systems, respectively; EI, exposure index; GP, general-public exposure; OE, occupational exposure; BR, basic restriction; NR, not reported; N/A, not applicable. TARO, an anatomical voxel adult human model developed by the National Institute of Information and Communications Technology, Japan. Where no directly comparable limit was available, ER was not calculated.

Electromagnetic exposure levels may differ substantially across age groups. Compared with adults, children and infants are shorter, placing the head and CNS closer to the high-field-strength regions near the transmitting coils of security-gate systems. In addition, the higher water content of pediatric tissues results in dielectric properties and electrical conductivities that differ from those of adult tissues. Under otherwise comparable exposure conditions, induced electric fields and current densities in localized tissues may therefore be higher in children and infants than in adults. By contrast, when changes in the effective cross-sectional area exposed to the field are limited, variations in posture appear to exert a relatively minor influence on whole-body-averaged SAR (152, 153).

A comparative analysis of adults and children aged 5 and 10 years reported that the maximum 1-cm^2^-averaged induced current density in the brain of the 5-year-old model reached 98.93 mA/m^2^, approximately 5.6 times the corresponding value in the adult model (17.63 mA/m^2^), and exceeded the public-exposure restriction specified by ICNIRP (154). A subsequent simulation study further reported that the maximum induced current density in the CNS of children aged 5–7 years reached 1.2 A/m^2^, compared with 0.8 A/m^2^ in adults. The corresponding peak SAR values were 0.0006 and 0.0005 W/kg, respectively (3).

Maternal and fetal dosimetry changes with gestational age because maternal geometry, fetal volume and position, and amniotic-fluid distribution evolve throughout pregnancy. The relatively high conductivity of amniotic fluid may contribute to localized current concentration near fetal tissues, while field-incidence direction and fetal position can further modify local exposure. Pregnancy assessments should therefore use gestational-age-specific anatomical models and account for fetal position and field orientation (135, 151).

Systematic numerical simulations have been conducted using anatomical models representing pregnant women and fetuses from the first to the ninth month of gestation (151). During early and mid-pregnancy, corresponding to gestational months 1–5, the calculated exposure metrics for maternal and fetal tissues remained within the restrictions specified by ICNIRP and IEEE. During gestational months 6–9, however, increases in fetal volume and changes in the geometry of the amniotic cavity resulted in higher localized induced current densities. Calculated values in certain regions exceeded the basic restrictions specified by ICNIRP while remaining within the corresponding limits defined by IEEE.

Twin pregnancies exhibit more complex anatomical structures and electromagnetic-coupling patterns than singleton pregnancies. High-resolution twin-fetus models at 31 and 32 weeks of gestation have been developed and compared with singleton models at the corresponding gestational ages (135). Under low-frequency uniform magnetic-field exposure, the twin models exhibited higher exposure levels than the singleton models. This difference may be attributable to the relative positions of the fetuses, the interfaces between adjacent fetal tissues, and localized electromagnetic-field interactions. Fetal number may therefore represent an additional dosimetric determinant in pregnancy-specific exposure assessment.

To facilitate quantitative interpretation across different security-gate technologies, representative experimental and numerical exposure data were normalized to the corresponding ICNIRP or IEEE limits. Table 7 reports the exposure ratio (ER = exposure/limit) and safety margin (SM = limit/exposure) only when the exposure metric and applicable limit are directly comparable.

**Table 7:** Quantitative comparison of representative exposure metrics with ICNIRP/IEEE limits and safety margins.

Device/exposure scenario	Frequency	Exposure metric	Reported exposure	Applicable limit	ER	SM	Interpretation	References
WTMD; direct field mapping of three commercial systems	WTMD-A: 3–14 kHz WTMD-B/C: 200–250 Hz	Measured *H*	21.3–27.9 A/m RMS	—	—	—	Direct measurements; ER/SM not derived for complex waveforms	(4)
WTMD; pregnant-woman model	940 Hz	*B*_avg_/*B_max_*	9.92/29.4 μT	ICNIRP: 6.25 μT; IEEE: 730 μT	1.59×/0.040×	0.63×/24.8×	ICNIRP RL exceeded; IEEE limit satisfied	(151)
WTMD; month-8 fetal model	940 Hz	Induced *J*/internal *E*	3.09 mA/m^2^/16.61 mV/m	ICNIRP: 2 mA/m^2^; IEEE: 277 mV/m	1.55×/0.060×	0.65×/16.7×	ICNIRP BR exceeded; IEEE BR satisfied	(151)
EAS (EM)	14 kHz	*B*_IEC_/induced *E*	111 μT/0.88 V/m	ICNIRP: 27 μT/1.9 V/m	4.11×/0.46×	0.24×/2.16×	External RL exceeded; internal BR satisfied	(53)
EAS (AM); adult female, worst-case posture	58 kHz	Maximum exposure index (EI)	22.5 (GP)/4.5 (OE)	EI = 1.0	22.5×/4.5×	0.044×/0.22×	BR exceeded in worst-case posture	(175)
EAS (RF); adult/child models	10 MHz	Induced *J*/SAR_₁₀g_	1.16 A/m^2^/5.53 × 10^−3^ W/kg	ICNIRP: 20 A/m^2^/2 W/kg	0.058×/0.0028×	17.2×/362×	BR satisfied	(3)
EAS; 5-year-old model	30 kHz	Brain *J*_1cm_^2^	98.93 mA/m^2^	ICNIRP: 60 mA/m^2^	1.65×	0.61×	BR exceeded	(154)
EAS (MW); direct field measurement	NR	Measured *E*	Up to 23.8 V/m	—	—	—	Insufficient data for matched safety margin	(142)
RFID; physical anti-theft gate	13.56 MHz	Measured *H*	68.3 mA/m	ICNIRP: 73 mA/m	0.94×	1.07×	Below RL	(111)
RFID; metallic implant, 5-cm worst-case model	125 kHz	*J*_1cm_^2^	3.30 A/m^2^	GP BR: 0.25 A/m^2^	13.2×	0.076×	BR exceeded under worst-case conditions	(159)
RFID; metallic implant, 5-cm worst-case model	13.56 MHz	SAR_₁₀g_	7.31 W/kg	GP BR: 2 W/kg	3.66×	0.27×	BR exceeded under worst-case conditions	(159)
Active MMW whole-body security scanner	30–100 GHz	Peak incident power density	1.0 kW/m^2^	ICNIRP: 10 kW/m^2^	0.10×	10.0×	Below limit	(176)

ER, exposure ratio (reported exposure/applicable limit); SM, safety margin (applicable limit/reported exposure = 1/ER). Both are dimensionless and are expressed as fold values (×). ER > 1 (SM < 1) indicates exceedance of the applicable limit, whereas ER < 1 (SM > 1) indicates a value below the limit. B, magnetic flux density; B_IEC_, IEC-defined 45-point average magnetic flux density; E, electric-field strength; H, magnetic-field strength; J, induced current density; J₁cm^2^, induced current density averaged over a 1-cm^2^ cross-section; SAR_10g_, specific absorption rate averaged over 10 g of contiguous tissue; EI, exposure index; RL, reference level; BR, basic restriction; GP, general public; OE, occupational exposure; RMS, root mean square; NR, not reported. Quantitative comparisons were made only when the reported metric, frequency, population category, and averaging basis were compatible with the applicable limit. Safety margins represent compliance margins rather than probabilities of adverse health effects. Worst-case numerical results apply only to the specified modeled conditions and should not be generalized to routine passage.

The comparisons show that compliance depends on frequency, exposure metric, exposure geometry, and population. Exceedance of an external reference level does not necessarily imply exceedance of an internal basic restriction, whereas selected susceptible-population or worst-case scenarios may exceed basic restrictions.

### 8.3 Numerical simulation results for electromagnetic interactions between medical devices and electromagnetic security gates

Susceptibility of implantable medical devices to security-gate EMI is frequency dependent (155). A quantitative relationship between ambient magnetic flux density and induced device voltage has been developed for AIMD assessment (11). The model considers a conservative unipolar pacing configuration, in which the pacemaker housing, lead, and cardiac tissue form an approximately semicircular loop. When a uniform time-varying magnetic field is perpendicular to the loop plane, the magnetic flux is given by Equation 1:

$$\Phi (t)=B_{\text{peak}}\cdot A_{\mathrm{eff}}\cdot \sin(2\mathrm{\pi ft})$$ (1)

According to Faraday’s law, the induced electromotive force—and therefore the voltage at the pacemaker sensing port—is determined by the time derivative of magnetic flux. This yields linear relationships between zero-to-peak magnetic flux density and peak sensing-port voltage (11, 101, 102). This yields the relationship shown in Equation 2:

$$U_{\mathrm{pp}}=2{|\frac{\mathrm{d\Phi}(t)}{\mathrm{dt}}|}_{\max}=4\mathrm{\pi f}A_{\mathrm{eff}}B_{\text{peak}}$$ (2)

Combining these relationships with experimentally measured frequency-dependent immunity thresholds allows the maximum permissible magnetic flux density, *B*_max_, to be estimated using Equation 3. For pulsed or amplitude-modulated security-gate fields, zero-to-peak magnetic flux density should therefore be considered rather than the root-mean-square value alone:

$$B_{\max}=\frac{U_{\mathrm{pp}\_\text{threshold}}}{4\mathrm{\pi f}A_{\mathrm{eff}}}$$ (3)

Here, *B*_peak_ is the zero-to-peak magnetic flux density (T), *U*_pp_ the peak-to-peak voltage at the pacemaker sensing port (V), *f* the operating frequency (Hz), and *A*_eff_ the effective induction area. For the conservative unipolar configuration, *A*_eff_ is set to 225 cm^2^ (0.0225 m^2^), representing the approximate maximum semicircular area formed by a left-pectorally implanted pacemaker lead and its return path in a large adult (11). *U*_pp_threshold_ denotes the experimentally determined voltage-immunity threshold.

Voltages induced between the pacemaker housing and the distal end of the ventricular lead were reported to be approximately 300 mV and 1 mV under RF and EM exposure conditions, respectively (156). These relatively low induced voltages were insufficient to cause severe pacemaker malfunction. High-resolution average-body-size and obesity models were used to evaluate induced voltages in pacemakers implanted in the left pectoral region, right pectoral region, and abdomen (157). The maximum interference voltage was approximately 10 mV, remaining well below the immunity levels specified by the applicable medical-device safety standards. The maximum interference voltage in the obesity model was 7.9 mV, approximately 21% lower than that in the average-body-size model, suggesting that thicker body tissues may attenuate electromagnetic coupling to some extent.

A sacral neurostimulator was also evaluated using an exposure simulator, and the measured induced-voltage waveforms were applied to a computational model of spinal-nerve axons (158). The results indicated that, even when the pulse generator was switched off, energy directly coupled into the lead could be sufficient to evoke unintended action potentials in nearby nerve axons.

Passive metallic implants may concentrate current near their edges, increasing induced current density and specific absorption rate (SAR) in adjacent tissues and thereby intensifying localized exposure (159). A refined framework for calculating localized electromagnetic exposure was subsequently established using the weighted peak method in the time domain (WPM-TD) (160). Building on this framework, low-frequency magnetic-field exposure in the hand and forearm was simulated in the presence of commonly used metallic implants (161). The results indicated that edge effects and pronounced conductivity contrasts between the implant and surrounding tissues can substantially amplify the average induced electric-field strength in tissues adjacent to the implant under otherwise identical external-field conditions.

### 8.4 Critical appraisal of numerical evidence

Within the framework of Section 2.3, numerical evidence was evaluated in two domains: human dosimetry and numerical or analytical assessment of medical-device and implant interactions. Equivalent-source and human-body-equivalent antenna models support these analyses but were not treated as a separate evidence domain because they represent exposure sources rather than independent safety endpoints.

Human dosimetry studies consistently indicate that anatomy can substantially influence localized exposure. Pediatric models may exhibit higher induced current densities than adult models, while gestational age, fetal position, and fetal number can modify maternal and fetal dosimetry (3, 135, 151–154). These simulations provide information that is difficult to obtain experimentally. Quantitative estimates, however, depend on model resolution, tissue electrical properties, source representation, field orientation, geometry, and numerical method. Comparison with exposure restrictions is further complicated by differences in dosimetric quantities and guideline frameworks. Some studies also use uniform or simplified source fields rather than the highly non-uniform fields of actual security gates, and experimental validation remains limited. Human numerical dosimetry was therefore rated as moderate in strength: the principal determinants and exposure trends are reasonably consistent, but model uncertainty and limited validation constrain quantitative generalization.

Numerical and analytical studies of medical-device and implant interactions link external fields to induced voltages, currents, and localized tissue exposure. Pacemaker models relate ambient magnetic flux density to sensing-port voltage and have examined the effects of implantation site and body habitus (11, 156, 157). Studies of neurostimulators and passive implants further show that coupling through leads or conductivity discontinuities can produce localized responses not evident from external-field measurements alone (158–161). However, the evidence base is comparatively small. Several analyses rely on conservative assumptions, simplified lead geometries, fixed coupling areas, or limited device configurations, and independent experimental validation remains sparse.

This body of numerical and analytical evidence was therefore rated as low in strength. The rating reflects limited directly comparable studies, device-specific assumptions, and insufficient independent replication rather than poor technical quality of individual studies. Current models are useful for identifying plausible coupling mechanisms and conservative exposure bounds, but broader conclusions require validation across contemporary implant designs, realistic source configurations, and standardized exposure conditions.

## 9 Conclusions and future directions

Electromagnetic security gates span technologies from low frequencies to millimeter waves and therefore produce markedly different exposure patterns and relevant dosimetric endpoints. Across the available experimental and numerical evidence, exposure is generally highest near transmitting antennas or gate panels and decreases with distance. Routine passage typically results in lower exposure than prolonged close-range or occupational scenarios (162), although selected susceptible-population and worst-case configurations may approach or exceed applicable restrictions. Medical-device interactions are likewise dependent on frequency, waveform, distance, dwell time, and device configuration, with low-frequency pulsed or modulated exposure warranting particular attention.

Under the structured appraisal framework used in this review, experimental field characterization, experimental interactions with active medical devices, human numerical dosimetry, and broader ELF/RF epidemiology were rated as moderate-strength bodies of evidence. Direct epidemiological evidence specific to security-gate exposure and numerical/analytical evidence concerning medical-device and implant interactions were rated as low in strength, primarily because of the limited number of directly relevant studies, heterogeneous exposure conditions, and insufficient independent validation or replication. Broader ELF/RF epidemiological evidence provides contextual rather than directly transferable evidence for security-gate-specific exposure. No evidence domain met the criteria for high-strength evidence, underscoring the need for standardized exposure protocols, improved validation, and independently replicated studies.

Despite these advances, several methodological and practical gaps remain.

1 Anatomical modeling and dosimetric accuracy. Conventional low-resolution voxel models may generate numerical artifacts and non-physical local field enhancements, particularly near tissue interfaces and geometrically complex structures. Future studies should increasingly employ high-fidelity, heterogeneous anatomical models that incorporate fine-scale structures, including microvascular networks and complex neural topologies (128).
2 Time-domain exposure assessment. Conventional steady-state measurements under approximately uniform-field conditions are insufficient to characterize highly dynamic emissions, particularly those generated by pulsed or amplitude-modulated security-gate systems (130, 160). Future measurement frameworks should extend beyond purely frequency-domain analysis toward time-domain assessment. Robust nonlinear filtering methods are needed to distinguish transient exposure peaks from background noise while maintaining numerical stability.
3 Exposure assessment for susceptible populations and individuals with passive implants. Localized current-concentration effects in maternal and fetal tissues, edge-related field enhancement near passive metallic orthopedic implants (163), and the comparatively elevated exposure levels reported for infants and children indicate that reliance on a single standard-adult reference model may be insufficient. More targeted safety constraints and assessment procedures should be developed for susceptible populations, taking into account anatomical differences, gestational age, fetal number, implant geometry, and exposure configuration.
4 Dosimetry of active millimeter-wave screening systems. The electromagnetic energy generated by active millimeter-wave security-screening systems is deposited predominantly in superficial tissues. However, quantitative assessments of localized absorption and potential amplification effects within microscale superficial tissue structures remain limited (114). Further investigation is required to characterize these near-surface exposure patterns and establish appropriate evaluation metrics.

Future work should combine realistic source characterization, validated anatomical models, standardized assessment protocols, and independent replication, while strengthening occupational and susceptible-population assessment and evidence-based risk communication. Such efforts will support the safe deployment of electromagnetic security technologies (164–166).

## Glossary

AIMD: active implantable medical device
AM: acousto-magnetic
ARPANSA: Australian Radiation Protection and Nuclear Safety Agency
BR: basic restriction
CAGR: compound annual growth rate
CDRH: Center for Devices and Radiological Health
CIED: cardiac implantable electronic device
CNS: central nervous system
EAS: electronic article surveillance
EHS: electromagnetic hypersensitivity
EI: exposure index
ELF: extremely low frequency
EM: electromagnetic
EMC: electromagnetic compatibility
EMF: electromagnetic field
EMI: electromagnetic interference
EN: European Standard
ER: exposure ratio
FCC: Federal Communications Commission
FDA: U.S. Food and Drug Administration
FDTD: finite-difference time-domain
FEM: finite-element method
GP: general public
HF: high frequency
ICD: implantable cardioverter-defibrillator
ICNIRP: International Commission on Non-Ionizing Radiation Protection
IEC: International Electrotechnical Commission
IEEE: Institute of Electrical and Electronics Engineers
ISED: Innovation, Science and Economic Development Canada
ISO: International Organization for Standardization
LC: inductor–capacitor
LF: low frequency
MDR: Medical Device Reporting
MIMO: multiple-input multiple-output
MMW: millimeter wave
MW: microwave
N/A: not applicable
NR: not reported
OE: occupational exposure
PM: pacemaker
PNS: peripheral nervous system
RF: radio frequency
RFID: radio-frequency identification
RL: reference level
RMS: root mean square
SAR: specific absorption rate
SAR10g: specific absorption rate averaged over 10 g of contiguous tissue
SARWB: whole-body-averaged specific absorption rate
SM: safety margin
SPFD: scalar-potential finite-difference method
SRD: short-range device
3D-IBIEM: three-dimensional indirect boundary integral equation method
Tx: transmitting/transmitter
Rx: receiving/receiver
UHF: ultra-high frequency
WPM-TD: weighted peak method in the time domain
WTMD: walk-through metal detector

## References

[1] Boivin W Coletta J Kerr L. Characterization of the magnetic fields around walk-through and hand-held metal detectors. Health Phys. (2003) 84:582–93. doi: 10.1097/00004032-200305000-00003, 12747477

[2] Roivainen P Eskelinen T Jokela K Juutilainen J. Occupational exposure to intermediate frequency and extremely low frequency magnetic fields among personnel working near electronic article surveillance systems. Bioelectromagnetics. (2014) 35:245–50. doi: 10.1002/bem.21850, 24615825

[3] Martínez-Búrdalo M Sanchis A Martín A Villar R. Comparison of SAR and induced current densities in adults and children exposed to electromagnetic fields from electronic article surveillance devices. Phys Med Biol. (2010) 55:1041–55. doi: 10.1088/0031-9155/55/4/009, 20090190

[4] Guag J Addissie B Witters D. Personal medical electronic devices and walk-through metal detector security systems: assessing electromagnetic interference effects. Biomed Eng Online. (2017) 16:33. doi: 10.1186/s12938-017-0328-9, 28320451 PMC5359895

[5] U.S. Food and Drug Administration. Guidance for Industry: Labeling for Electronic Anti-Theft Systems. Rockville, MD: U.S. Food and Drug Administration, Center for Devices and Radiological Health (2000).

[6] Singh S Kapoor N. Health implications of electromagnetic fields, mechanisms of action, and research needs. Adv Biol. (2014) 2014:1–24. doi: 10.1155/2014/198609

[7] Liu L Huang B Lu Y Zhao Y Tang X Shi Y. Interactions between electromagnetic radiation and biological systems. iScience. (2024) 27:109201. doi: 10.1016/j.isci.2024.109201, 38433903 PMC10906530

[8] Bhatt BP Rathod JM Parmar YD A comparative study of electronic article surveillance (EAS) system. Proceedings of Second International Conference on Signals, Systems & Automation (ICSSA-11) (2011). 474–477.

[9] Want R. An introduction to RFID technology. IEEE Pervasive Comput. (2006) 5:25–33. doi: 10.1109/MPRV.2006.2

[10] Sheen DM McMakin DL Hall TE. Three-dimensional millimeter-wave imaging for concealed weapon detection. IEEE Trans Microw Theory Tech. (2001) 49:1581–92. doi: 10.1109/22.942570

[11] Irnich W. Electronic security systems and active implantable medical devices. Pacing Clin Electrophysiol. (2002) 25:1235–58. doi: 10.1046/j.1460-9592.2002.01235.x, 12358176

[12] Conners GP. Diagnostic uses of metal detectors: a review. Int J Clin Pract. (2005) 59:946–9. doi: 10.1111/j.1742-1241.2005.00456.x 16033617

[13] Dawson S. Consumer responses to electronic article surveillance alarms. J Retail. (1993) 69:353–62. doi: 10.1016/0022-4359(93)90011-7

[14] Haas DJ. Electronic security screening: its origin with aviation security 1968–1973. J Appl Secur Res. (2010) 5:460–532. doi: 10.1080/19361610.2010.505778

[15] Luo Q He Y Sun Y. Real-time fault detection and diagnosis system for analog and mixed-signal circuits of acousto–magnetic EAS devices. IEEE Des Test. (2016) 33:77–90. doi: 10.1109/MDAT.2015.2480714

[16] Vanderpool J Giles OS. "Managing EAS system and medical implant interactions". In: 2002 IEEE International Symposium on Electromagnetic Compatibility, vol. 2 (2002) Piscataway, NJ, USA: IEEE. p. 925–30.

[17] Landt J. The history of RFID. IEEE Potentials. (2005) 24:8–11. doi: 10.1109/MP.2005.1549751

[18] Chen H Lee S Rao R Slamani M Varshney P. Imaging for concealed weapon detection: a tutorial overview of development in imaging sensors and processing. IEEE Signal Process Mag. (2005) 22:52–61. doi: 10.1109/MSP.2005.1406480

[19] Bian L Li D Wang S Teng C Wu J Liu H . Towards large-scale single-shot millimeter-wave imaging for low-cost security inspection. Nat Commun. (2024) 15:6459. doi: 10.1038/s41467-024-50288-y, 39085225 PMC11291932

[20] Markets and Markets. Security Screening Market Size, Share & Trends, 2025 To 2030. Pune, India: Markets and Markets (2025).

[21] Markets and Markets. Electronic Article Surveillance Market by Component (Tags, Antennas & Detachers), Technology, End User (Apparel & Fashion Accessories Stores, Supermarket & Mass Merchandise Stores, Drug & Health Product Stores), and Geography-Global Forecast to 2036. Pune, India: Markets and Markets (2025).

[22] Data Bridge Market Research. Global RFID Portals for Doorways and Corridors Market. Pune, India: Data Bridge Market Research (2025).

[23] Persistence Market Research. Millimeter Wave Body Scanner Market. London, UK: Persistence Market Research (2026).

[24] Nevoit G Potyazhenko M Mintser O Jarusevicius G Vainoras A. Human bioelectromagnetism and the environment: introduction to the problem. Appl Sci. (2026) 16:3627. doi: 10.3390/app16083627

[25] Nguyen TA, editor. Advances in Bioelectromagnetism: Innovations and Applications in Healthcare. 1st ed. Cambridge, MA: Academic Press (2025).

[26] Chen XL Benkler S Chavannes N Kuster N Full human body exposure assessment in low frequency electromagnetic fields 2010 Asia-Pacific International Symposium on Electromagnetic Compatibility; (2010) 366–369.

[27] Lin JC. Reassessing laboratory results of low-frequency electromagnetic field exposure of cells in culture [telecommunications health and safety]. IEEE Antennas Propag Mag. (2014) 56:227–9. doi: 10.1109/MAP.2014.6821789

[28] Lin JC. Human exposure to RF, microwave, and millimeter-wave electromagnetic radiation [health effects]. IEEE Microw Mag. (2016) 17:32–6. doi: 10.1109/MMM.2016.2538540

[29] Yamazaki K Taki M Ohkubo C. Safety assessment of human exposure to intermediate frequency electromagnetic fields. Electr Eng Jpn. (2016) 197:3–11. doi: 10.1002/eej.22884

[30] Hirata A Diao Y Onishi T Sasaki K Ahn S Colombi D . Assessment of human exposure to electromagnetic fields: review and future directions. IEEE Trans Electromagn Compat. (2021) 63:1619–30. doi: 10.1109/TEMC.2021.3109249

[31] Nikolopoulos D. Electromagnetic radiation and human environment: editorial. Appl Sci (Basel). (2026) 16:5051. doi: 10.3390/app16105051

[32] Gabriel C Gabriel S Corthout E. The dielectric properties of biological tissues: I. Literature survey. Phys Med Biol. (1996) 41:2231–49. doi: 10.1088/0031-9155/41/11/001, 8938024

[33] Gabriel S Lau RW Gabriel C. The dielectric properties of biological tissues: II. Measurements in the frequency range 10 Hz to 20 GHz. Phys Med Biol. (1996) 41:2251–69. doi: 10.1088/0031-9155/41/11/002, 8938025

[34] Gabriel S Lau RW Gabriel C. The dielectric properties of biological tissues: III. Parametric models for the dielectric spectrum of tissues. Phys Med Biol. (1996) 41:2271–93. doi: 10.1088/0031-9155/41/11/003, 8938026

[35] Razek A. Biological and medical disturbances due to exposure to fields emitted by electromagnetic energy devices—a review. Energies. (2022) 15:4455. doi: 10.3390/en15124455

[36] Judakova Z Janousek L Radil R Carnecka L. Low-frequency magnetic field exposure system for cells electromagnetic biocompatibility studies. Appl Sci. (2022) 12:6846. doi: 10.3390/app12146846

[37] Bodewein L Schmiedchen K Dechent D Stunder D Graefrath D Winter L . Systematic review on the biological effects of electric, magnetic and electromagnetic fields in the intermediate frequency range (300 Hz to 1 MHz). Environ Res. (2019) 171:247–59. doi: 10.1016/j.envres.2019.01.015, 30690271

[38] Lin JC. Health and safety practices and policies concerning human exposure to RF/microwave radiation. Front Public Health. (2025) 13:1619781. doi: 10.3389/fpubh.2025.1619781, 40761932 PMC12318757

[39] Meyer F Bitsch A Forman HJ Fragoulis A Ghezzi P Henschenmacher B . The effects of radiofrequency electromagnetic field exposure on biomarkers of oxidative stress in vivo and in vitro: a systematic review of experimental studies. Environ Int. (2024) 194:108940. doi: 10.1016/j.envint.2024.108940, 39566441

[40] Piszczek P Wójcik-Piotrowicz K Guzdek P Gil K Kaszuba-Zwoińska J. Protein expression changes during phagocytosis influenced by low-frequency electromagnetic field exposure. Int J Biol Macromol. (2022) 217:481–91. doi: 10.1016/j.ijbiomac.2022.07.080, 35841960

[41] Panagopoulos DJ. Comparing DNA damage induced by mobile telephony and other types of man-made electromagnetic fields. Rev Mutat Res. (2019) 781:53–62. doi: 10.1016/j.mrrev.2019.03.003, 31416578

[42] Kenny RPW Johnson EE Adesanya AM Richmond C Beyer F Calderon C . The effects of radiofrequency exposure on male fertility: a systematic review of human observational studies with dose–response meta-analysis. Environ Int. (2024) 190:108817. doi: 10.1016/j.envint.2024.108817 38880061

[43] Johnson EE Kenny RPW Adesanya AM Richmond C Beyer F Calderon C . The effects of radiofrequency exposure on adverse female reproductive outcomes: a systematic review of human observational studies with dose–response meta-analysis. Environ Int. (2024) 190:108816. doi: 10.1016/j.envint.2024.108816, 38880062

[44] Kaplan S Deniz OG Önger ME Türkmen AP Yurt KK Aydın I . Electromagnetic field and brain development. J Chem Neuroanat. (2016) 75:52–61. doi: 10.1016/j.jchemneu.2015.11.005, 26686296

[45] Gross J Junghöfer M Wolters C. Bioelectromagnetism in human brain research: new applications, new questions. Neuroscientist. (2023) 29:62–77. doi: 10.1177/10738584211054742, 34873945 PMC9902961

[46] Pitron V Haanes JV Hillert L Köteles FG Léger D Lemogne C . Electrohypersensitivity is always real. Environ Res. (2023) 218:114840. doi: 10.1016/j.envres.2022.114840, 36463993

[47] Röösli M Dongus S Jalilian H Eyers J Esu E Oringanje CM . The effects of radiofrequency electromagnetic fields exposure on tinnitus, migraine and non-specific symptoms in the general and working population: a systematic review and meta-analysis on human observational studies. Environ Int. (2024) 183:108338. doi: 10.1016/j.envint.2023.108338, 38104437

[48] Bosch-Capblanch X Esu E Oringanje CM Dongus S Jalilian H Eyers J . The effects of radiofrequency electromagnetic fields exposure on human self-reported symptoms: a systematic review of human experimental studies. Environ Int. (2024) 187:108612. doi: 10.1016/j.envint.2024.108612 38640611

[49] Kheifets L Shimkhada R. Childhood leukemia and EMF: review of the epidemiologic evidence. Bioelectromagnetics. (2005) 26:S51–9. doi: 10.1002/bem.20139, 16059924

[50] Singh N Mathur R Behari J. Electromagnetic fields and cancer. IETE Tech Rev. (1997) 14:149–51. doi: 10.1080/02564602.1997.11416664

[51] Kheifets LI Matkin CC. Industrialization, electromagnetic fields, and breast cancer risk. Environ Health Perspect. (1999) 107:145–54. doi: 10.1289/ehp.99107s1145, 10229714 PMC1566357

[52] Karipidis K Baaken D Loney T Blettner M Brzozek C Elwood M . The effect of exposure to radiofrequency fields on cancer risk in the general and working population: a systematic review of human observational studies – part I: Most researched outcomes. Environ Int. (2024) 191:108983. doi: 10.1016/j.envint.2024.108983, 39241333

[53] Ikuyo M Esaki K Aimoto A Wake K Yamaguchi-Sekino S Kojimahara N . Measurement and exposure assessment of intermediate frequency magnetic fields from electronic article surveillance (EAS) gates in libraries. Front Public Health. (2022) 10:871134. doi: 10.3389/fpubh.2022.871134, 35646792 PMC9133885

[54] Yamaguchi-Sekino S Taki M Ikuyo M Esaki K Aimoto A Wake K . Assessment of combined exposure to intermediate-frequency electromagnetic fields and pulsed electromagnetic fields among library workers in Japan. Front Public Health. (2022) 10:870784. doi: 10.3389/fpubh.2022.870784, 35968480 PMC9366663

[55] Khan MW Roivainen P Herrala M Tiikkaja M Sallmén M Hietanen M . A pilot study on the reproductive risks of maternal exposure to magnetic fields from electronic article surveillance systems. Int J Radiat Biol. (2018) 94:902–8. doi: 10.1080/09553002.2018.1439197, 29419348

[56] Amoon AT Swanson J Magnani C Johansen C Kheifets L. Pooled analysis of recent studies of magnetic fields and childhood leukemia. Environ Res. (2022) 204:111993. doi: 10.1016/j.envres.2021.111993, 34481821

[57] Brabant C Geerinck A Beaudart C Tirelli E Geuzaine C Bruyère O. Exposure to magnetic fields and childhood leukemia: a systematic review and meta-analysis of case-control and cohort studies. Rev Environ Health. (2023) 38:229–53. doi: 10.1515/reveh-2021-0112, 35302721

[58] Huss A Spoerri A Egger M Kromhout H Vermeulen R. Occupational extremely low frequency magnetic fields (ELF-MF) exposure and hematolymphopoietic cancers – Swiss National Cohort analysis and updated meta-analysis. Environ Res. (2018) 164:467–74. doi: 10.1016/j.envres.2018.03.022, 29587222

[59] Litvak E Foster KR Repacholi MH. Health and safety implications of exposure to electromagnetic fields in the frequency range 300 Hz to 10 MHz. Bioelectromagnetics. (2002) 23:68–82. doi: 10.1002/bem.99, 11793407

[60] Potenza L Cucchiarini L Vetrano F Dacha M. Electric and magnetic fields as possible risk factors for human health. Int J Risk Assess Manag. (2005) 5:292–310. doi: 10.1504/ijram.2005.007173, 35009967

[61] Przystupa K Pohrebennyk V Mitryasova O Kochan O The influence of electromagnetic field on human health 2019 Applications of Electromagnetics in Modern Engineering and Medicine (PTZE); (2019). 139–42.

[62] Zradziński P. A comparison of ICNIRP and IEEE guidelines to evaluate low frequency magnetic field localised exposure 2016 17th International Conference Computational Problems of Electrical Engineering (CPEE). (2016):1–4.

[63] IEEE IEEE Standard for Safety Levels with Respect to Human Exposure to Radio Frequency Electromagnetic Fields, 3 kHz to 300 GHz. IEEE Std C95.1-1991, (1991). 1–76.

[64] IEEE IEEE Standard for Safety Levels with Respect to Human Exposure to Radio Frequency Electromagnetic Fields, 3 kHz to 300 GHz. IEEE Std C95.1-1999 Edition. (1999). 1–83.

[65] IEEE IEEE Standard for Safety Levels With Respect to Human Exposure to Electromagnetic Fields, 0–3 kHz. IEEE Std C95.6-2002. (2002). 1–64.

[66] IEEE IEEE Standard for Safety Levels with Respect to Human Exposure to Radio Frequency Electromagnetic Fields, 3 kHz to 300 GHz. IEEE Std C951-2005 (Revision of IEEE Std C95.1-2005). (2006). 1–238.

[67] IEEE IEEE Standard for Safety Levels with Respect to Human Exposure to Electric, Magnetic, and Electromagnetic Fields, 0 Hz to 300 GHz. IEEE Std C95, 1–2019 (Revision of IEEE Std C95, 1–2005/ Incorporates IEEE Std C95.1-2019) (2019) 1–312

[68] ICNIRP. Guidelines for limiting exposure to time-varying electric, magnetic, and electromagnetic fields (up to 300 GHz). Health Phys. (1998) 74:494–522. 9525427

[69] ICNIRP. Guidelines for limiting exposure to time-varying electric and magnetic fields (1 Hz to 100 kHz). Health Phys. (2010) 99:818–36. doi: 10.1097/HP.0b013e3181f06c86 21068601

[70] ICNIRP. Guidelines for limiting exposure to electromagnetic fields (100 kHz to 300 GHz). Health Phys. (2020) 118:483–524. doi: 10.1097/hp.0000000000001210, 32167495

[71] Joseph W Vermeeren G Verloock L Goeminne F. In situ magnetic field exposure and ICNIRP-based safety distances for electronic article surveillance systems. Radiat Prot Dosim. (2012) 148:420–7. doi: 10.1093/rpd/ncr206, 21613266

[72] ICNIRP. ICNIRP statement related to the use of security and similar devices utilizing electromagnetic fields. Health Phys. (2004) 87:187–96. doi: 10.1097/00004032-200408000-00007 15257219

[73] IEC. Evaluation of human Exposure to Electromagnetic fields from short range Devices (SRDs) in Various Applications over the Frequency range 0 GHz to 300 GHz—Part 1: Fields Produced by Devices Used for Electronic Article Surveillance, radio Frequency Identification and similar Systems. IEC 62369-1: (2008) Geneva, Switzerland: International Electrotechnical Commission (IEC).

[74] European Committee for Electrotechnical Standardization (CENELEC). Product Standard for human Exposure to Electromagnetic fields from Devices Operating in the Frequency range 0 Hz to 300 GHz, Used in Electronic Article Surveillance (EAS), Radio Frequency Identification (RFID) and similar Applications. (2018). Brussels, Belgium: European Committee for Electrotechnical Standardization (CENELEC).

[75] Council of the European Union. Council Recommendation of 12 July 1999 on the Limitation of Exposure of the general public to Electromagnetic fields (0 Hz to 300 GHz) (1999/519/EC). Luxembourg: Official Journal of the European Communities (1999). p. 59–70.

[76] European Parliament and Council of the European Union. Directive 2013/35/EU of the European Parliament and of the Council of 26 June 2013 on the minimum Health and Safety Requirements Regarding the Exposure of Workers to the Risks Arising from Physical Agents (Electromagnetic fields) (20th Individual Directive within the Meaning of Article 16(1) of Directive 89/391/EEC) and Repealing Directive 2004/40/EC. Luxembourg: Official Journal of the European Union (2013). p. 1–21.

[77] Federal Communications Commission. Radiofrequency Radiation Exposure Requirements: 47 CFR §§ 1.1307 and 1.1310 Code of Federal Regulations. Washington, DC: Federal Communications Commission (2026).

[78] U.S. Food and Drug Administration. Electromagnetic Compatibility (EMC) of Medical Devices: Guidance for Industry and Food and Drug Administration Staff. Silver Spring, MD: Guidance for Industry and Food and Drug Administration Staff (2022).

[79] Health Canada. Limits of Human Exposure to Radiofrequency Electromagnetic Energy in the Frequency Range from 3 kHz to 300 GHz. Safety Code 6. Ottawa, ON: Health Canada (2015).

[80] Innovation, Science and Economic Development Canada. Radio Frequency (RF) Exposure Compliance of Radiocommunication Apparatus (All Frequency Bands). Radio Standards Specification RSS-102. Ottawa, ON: Innovation, Science and Economic Development Canada (2024).

[81] Australian Radiation Protection and Nuclear Safety Agency. Standard for Limiting Exposure to Radiofrequency Fields – 100 kHz to 300 GHz. Radiation Protection Series S-1. Yallambie, VIC: Australian Radiation Protection and Nuclear Safety Agency (2021).

[82] Ministry of Environmental Protection of the People's Republic of China, General Administration of Quality Supervision, Inspection and Quarantine of the People's Republic of China. Controlling Limits for Electromagnetic Environment (GB 8702–2014). Beijing: China Environmental Science Press (2014).

[83] World Health Organization. Establishing a Dialogue on Risks from Electromagnetic Fields. Handbook. Geneva: World Health Organization (2002).

[84] Copperman Y Zarfati D Laniado S. The effect of metal detector gates on implanted permanent pacemakers. Pacing Clin Electrophysiol. (1988) 11:1386–97. doi: 10.1111/j.1540-8159.1988.tb04985.x, 2462212

[85] Kolb C Schmieder S Lehmann G Zrenner B Karch MR Plewan A . Do airport metal detectors interfere with implantable pacemakers or cardioverter-defibrillators? J Am Coll Cardiol. (2003) 41:2054–9. doi: 10.1016/s0735-1097(03)00424-8, 12798581

[86] Kamineni S Legge S Ware H. Metallic orthopaedic implants and airport metal detectors. J Arthroplast. (2002) 17:62–5. doi: 10.1054/arth.2002.28726, 11805926

[87] Van Der Wal BCH Grimm B Heyligers IC. Airport detectors and orthopaedic implants. Acta Orthop Belg. (2005) 71:379–84. 16184989

[88] Ismail A Dancey A Titley OG. Prosthetic metal implants and airport metal detectors. Ann R Coll Surg Engl. (2013) 95:211–4. doi: 10.1308/003588413x13511609955977, 23827294 PMC4165247

[89] McIvor ME Reddinger J Floden E Sheppard RC Johnson D Becker GI . Study of pacemaker and implantable cardioverter defibrillator triggering by electronic article surveillance devices (SPICED TEAS). Pacing Clin Electrophysiol. (1998) 21:1847–61. doi: 10.1111/j.1540-8159.1998.tb00002.x 9793079

[90] Groh WJ Boschee SA Engelstein ED Miles WM Burton ME Foster PR . Interactions between electronic article surveillance systems and implantable cardioverter-defibrillators. Circulation. (1999) 100:387–92. doi: 10.1161/01.CIR.100.4.387, 10421599

[91] Gimbel JR Cox JW Jr. Electronic article surveillance systems and interactions with implantable cardiac devices: risk of adverse interactions in public and commercial spaces. Mayo Clin Proc. (2007) 82:318–22. doi: 10.4065/82.3.318, 17352368

[92] McIvor ME. Environmental electromagnetic interference from electronic article surveillance devices: interactions with an ICD. Pacing Clin Electrophysiol. (1995) 18:2229–30. doi: 10.1111/j.1540-8159.1995.tb04654.x, 8771140

[93] Eisenberg E Waisbrod H. Spinal cord stimulator activation by an antitheft device. Case report. J Neurosurg. (1997) 87:961–2. doi: 10.3171/jns.1997.87.6.0961, 9384412

[94] Seidman SJ Brockman R Lewis BM Guag J Shein MJ Clement WJ . In vitro tests reveal sample radiofrequency identification readers inducing clinically significant electromagnetic interference to implantable pacemakers and implantable cardioverter-defibrillators. Heart Rhythm. (2010) 7:99–107. doi: 10.1016/j.hrthm.2009.09.071, 20129290

[95] Pantchenko OS Seidman SJ Guag JW Witters DM Jr Sponberg CL. Electromagnetic compatibility of implantable neurostimulators to RFID emitters. Biomed Eng Online. (2011) 10:50. doi: 10.1186/1475-925x-10-50, 21658266 PMC3135567

[96] Kong S. Choi D. Oh H. Evaluation of human exposure to electromagnetic fields from RFID devices at 13.56 MHz 2008 Asia-Pacific Microwave Conference; (2008). 1–4. doi: 10.1109/APMC.2008.4958434

[97] Dodinot B Godenir J-P Costa AB. Electronic article surveillance: a possible danger for pacemaker patients. Pacing Clin Electrophysiol. (1993) 16:46–53. doi: 10.1111/j.1540-8159.1993.tb01534.x, 7681175

[98] Lucas EH Johnson D Mcelroy BP. The effects of electronic article surveillance systems on permanent cardiac pacemakers: an in vitro study. Pacing Clin Electrophysiol. (1994) 17:2021–6. doi: 10.1111/j.1540-8159.1994.tb03793.x, 7845811

[99] Mugica J Henry L Podeur H. Study of interactions between permanent pacemakers and electronic antitheft surveillance systems. Pacing Clin Electrophysiol. (2000) 23:333–7. doi: 10.1111/j.1540-8159.2000.tb06758.x, 10750133

[100] Tiikkaja M Aro AL Alanko T Lindholm H Sistonen H Hartikainen JEK . Electromagnetic interference with cardiac pacemakers and implantable cardioverter-defibrillators from low-frequency electromagnetic fields in vivo. EP Europace. (2013) 15:388–94. doi: 10.1093/europace/eus345, 23125355

[101] Chave AD Weidelt P. "The theoretical basis for electromagnetic induction". In: Chave AD Jones AG, editors. The Magnetotelluric Method: Theory and Practice. Cambridge: Cambridge University Press (2012). p. 19–49. doi: 10.1017/CBO9781139020138.003

[102] Everett ME Chave AD. On the physical principles underlying electromagnetic induction. Geophysics. (2019) 84:W21–32. doi: 10.1190/geo2018-0232.1

[103] Hustedt A Craig C Technical review and optimization of walk-through metal detector processes 2018 IEEE International Symposium on Technologies for Homeland Security (HST). (2018). 386–95. doi: 10.1109/THS.2018.8574196

[104] Skorupski J Uchroński P. A fuzzy model for evaluating metal detection equipment at airport security screening checkpoints. Int J Crit Infrastruct Prot. (2017) 16:39–48. doi: 10.1016/j.ijcip.2016.11.001

[105] Dhagat P Jander A Luo D. Metal detector coil arrangement for uniform internal and zero external sensitivity. J Appl Phys. (2008) 103:07E919. doi: 10.1063/1.2833757

[106] Marsh LA Ktistis C Järvi A Armitage DW Peyton AJ. Three-dimensional object location and inversion of the magnetic polarizability tensor at a single frequency using a walk-through metal detector. Meas Sci Technol. (2013) 24:045102. doi: 10.1088/0957-0233/24/4/045102

[107] Nelson CV Cooperman CB Schneider W Wenstrand DS Smith DG. Wide bandwidth time-domain electromagnetic sensor for metal target classification. IEEE Trans Geosci Remote Sens. (2001) 39:1129–38. doi: 10.1109/36.927425

[108] Kim JH Seong H-C Lee J-H Kim K Yu B Bang J. A study on efficient evaluation methods for electromagnetic exposure to the human body from electronic article surveillance systems. J Electr Eng Technol. (2025) 20:5711–24. doi: 10.1007/s42835-025-02374-4

[109] Ramos V Suárez OJ Santana VMF Rodríguez DSS Aguirre E Miguel-Bilbao SD . Electromagnetic characterization of UHF-RFID fixed reader in healthcare centers related to the personal and labor health. IEEE Access. (2022) 10:28614–30. doi: 10.1109/ACCESS.2022.3156477

[110] Ni LM Zhang D Souryal MR. RFID-based localization and tracking technologies. IEEE Wirel Commun. (2011) 18:45–51. doi: 10.1109/MWC.2011.5751295

[111] Senic D Poljak D Šarolić A. Simulation and measurements of electric and magnetic fields of an RFID loop antenna anti-theft gate system. J Commun Softw Syst. (2010) 6:133–40. doi: 10.24138/JCOMSS.V6I4.186

[112] Zulim I Senic D Poljak D Sarolic A Assessment of SAR in the human body exposed to an RFID loop antenna. SoftCOM 2012, 20th International Conference on Software, Telecommunications and Computer Networks; (2012). 1–5.

[113] Shao W Li Y. An efficient method for modelling millimeter-wave scan for security screening of humans. IEEE Trans Comput Imaging. (2024) 10:1616–25. doi: 10.1109/TCI.2024.3487393

[114] Sun X Salmon NA Miao J. Performance analysis of a walk-through portal Array for passive millimeter-wave 3-D imaging. IEEE Trans Instrum Meas. (2024) 73:1–15. doi: 10.1109/TIM.2024.3353863, 25079929

[115] Onojima S Arima Y Hirose A. Millimeter-wave security imaging using complex-valued self-organizing map for visualization of moving targets. Neurocomputing. (2014) 134:247–53. doi: 10.1016/j.neucom.2013.06.048

[116] Wang Z Chang T Cui HL. Review of active millimeter wave imaging techniques for personnel security screening. IEEE Access. (2019) 7:148336–50. doi: 10.1109/ACCESS.2019.2946736

[117] Ahmed SS. Microwave imaging in security — two decades of innovation. IEEE J Microw. (2021) 1:191–201. doi: 10.1109/JMW.2020.3035790

[118] Wang Z Tian X Chang T Xu W Cui H-L Guo Q . Walk-through millimeter wave imaging testbed based on double multistatic cross arrays. Opt Express. (2022) 30:30356–67. doi: 10.1364/OE.460558, 36242141

[119] Appleby R Anderton RN. Millimeter-wave and submillimeter-wave imaging for security and surveillance. Proc IEEE. (2007) 95:1683–90. doi: 10.1109/JPROC.2007.898832

[120] Brooker I Electromagnetic field exposure assessment for EAS, RFID and similar devices. Sixteenth International Wrocław Symposium and Exhibition, Electromagnetic Compatibility 2002; (2002). 1–6.

[121] European Committee for Electrotechnical Standardization (CENELEC). Evaluation of human Exposure to Electromagnetic fields from short range Devices (SRDs) in Various Applications over the Frequency range 0 GHz to 300 GHz—Part 1: Fields Produced by Devices Used for Electronic Article Surveillance, radio Frequency Identification and similar Systems. EN 62369-1:2009. Brussels, Belgium: CENELEC (2009).

[122] Nelson C. Kantor P. Nakamura B. Ricks B. Whytlaw R. Egan D. Experimental designs for testing metal detectors at a large sports stadium 2015 IEEE International Symposium on Technologies for Homeland Security (HST); (2015). 1–7.

[123] Paulter NG. Test methods to rigorously, reproducibly, and accurately measure the detection performance of walk-through metal detectors. J Test Eval. (2020) 48:1694–711. doi: 10.1520/jte20180220, 38497029 PMC10941206

[124] Paulter NG Larson DR Ely JA. Test object for accurate and reproducible measurement of the detection response of hand-worn and hand-held metal detectors. J Res Nat Inst Stand Technol. (2016) 121:401–19. doi: 10.6028/jres.121.019, 34434630 PMC7339660

[125] Vulević B Osmokrović P. Evaluation of uncertainty in the measurement of environmental electromagnetic fields. Radiat Prot Dosim. (2010) 141:173–7. doi: 10.1093/rpd/ncq158, 20511401

[126] ISO. ISO 14117:2019 Active Implantable Medical Devices—Electromagnetic Compatibility—EMC test Protocols for Implantable cardiac Pacemakers, Implantable Cardioverter Defibrillators and cardiac Resynchronization devices. (2019). Geneva, Switzerland: International Organization for Standardization (ISO).

[127] CENELEC. EN 50527–1:2016 Procedure for the Assessment of the Exposure to Electromagnetic fields of Workers Bearing Active Implantable Medical Devices—Part 1: General. (2016). Brussels, Belgium: European Committee for Electrotechnical Standardization (CENELEC).

[128] Zradziński P. Difficulties in applying numerical simulations to an evaluation of occupational hazards caused by electromagnetic fields. Int J Occup Saf Ergon. (2015) 21:213–20. doi: 10.1080/10803548.2015.1028233, 26323781 PMC4566884

[129] Gandhi OP Lazzi G Sun J Kang G Use of the impedance method for ICNIRP-related compliance testing of electronic article surveillance devices. In: IEEE Antennas and Propagation Society International Symposium; 16–21 July. (2000) Salt Lake City, UT, USA. 2 1092–1095. doi: 10.1109/APS.2000.875412

[130] Kang G Gandhi OP. Comparison of various safety guidelines for electronic article surveillance devices with pulsed magnetic fields. IEEE Trans Biomed Eng. (2003) 50:107–13. doi: 10.1109/TBME.2002.807318, 12617530

[131] Kainz W Casamento JP Ruggera PS Chan DD Witters DM. Implantable cardiac pacemaker electromagnetic compatibility testing in a novel security system simulator. IEEE Trans Biomed Eng. (2005) 52:520–30. doi: 10.1109/TBME.2004.843293, 15759582

[132] Orcutt N Gandhi OP. A 3-D impedance method to calculate power deposition in biological bodies subjected to time varying magnetic fields. IEEE Trans Biomed Eng. (1988) 35:577–83. doi: 10.1109/10.4590, 3049307

[133] Dawson TW De Moerloose J Stuchly MA. Comparison of magnetically induced ELF fields in humans computed by FDTD and scalar potential FD codes. Appl Comput Electromagn Soc J. (1996) 11:63–71.

[134] Qiang R Wu D Chen J Wang S Wilton D Kainz W. An efficient two-dimensional FDTD method for bio-electromagnetic applications. IEEE Trans Magn. (2006) 42:1391–4. doi: 10.1109/TMAG.2006.871947

[135] Zhang C Li C Yang L Hou W Du M Wu T . Assessment of twin fetal exposure to environmental magnetic and electromagnetic fields. Bioelectromagnetics. (2022) 43:160–73. doi: 10.1002/bem.22397, 35233784

[136] Im C-H Jung H-K. Numerical emulator for walk-through metal detectors using 3-D indirect boundary integral equation method. IEEE Trans Instrum Meas. (2005) 54:1166–70. doi: 10.1109/TIM.2005.847241

[137] Misakian M Casamento JP Laug B. Development of emulator for walk-through metal detectors. IEEE Trans Electromagn Compat. (2002) 44:486–9. doi: 10.1109/TEMC.2002.801775

[138] Seidman SJ Kainz W Casamento J Witters D. Electromagnetic compatibility testing of implantable neurostimulators exposed to metal detectors. Open Biomed Eng J. (2010) 4:63–70. doi: 10.2174/1874120701004010063, 20448818 PMC2864430

[139] Lee J. G. Byun J. K. Choi H. D. Cheon C. Y. Chung Y. S. Lee B. A new equivalent antenna model for human body exposed to 13.56 MHz RFID system 2008 IEEE Antennas and Propagation Society International Symposium; (2008). 1–4. doi: 10.1109/APS.2008.4619692

[140] Wu D Qiang R Chen J Kainz W Seidman S Safety evaluation of walk-through metal detectors 2005 International Symposium on Electromagnetic Compatibility, (2005) 3, 796–800. doi: 10.1109/ISEMC.2005.1513633

[141] Casamento J. Characterizing electromagnetic fields of common electronic article surveillance systems. Compliance Eng. (1999) 16:42–52.

[142] Harris C Boivin W Boyd S Coletta J Kerr L Kempa K . Electromagnetic field strength levels surrounding electronic article surveillance (EAS) systems. Health Phys. (2000) 78:21–7. doi: 10.1097/00004032-200001000-00005, 10608306

[143] Trulsson J Anger G Estenberg U. Assessment of magnetic fields surrounding electronic article surveillance systems in Sweden. Bioelectromagnetics. (2007) 28:664–6. doi: 10.1002/bem.20359, 17786978

[144] Estenberg U Anger G Trulsson J Kartläggning av exponering för magnetfält runt larmbågar och RFID-system. Stockholm, Sweden: Statens strålskyddsinstitut (2006).

[145] Piaseczny M. Abramowicz A. Directional antenna for EAS systems MIKON 2008-17th International Conference on Microwaves, Radar and Wireless Communications. (2008). 1–4.

[146] Joseph W. Verloock L. Vermeeren G. Goeminne F. Martens L. Assessment of field exposure by electronic article surveillance systems 33rd Annual Meeting of the Bioelectromagnetics Society (BEMS 2011), Proceedings Ghent, Belgium Ghent University, Department of Information Technology. (2011) Halifax, Canada

[147] U.S. Food and Drug Administration. Important Information on Anti-Theft and Metal Detector Systems and Pacemakers, ICDs, and Spinal Cord Stimulators. Rockville, MD: U.S. Food and Drug Administration (1998).

[148] Harthorne JW. Pacemakers and store security devices. Cardiol Rev. (2001) 9:10–7. doi: 10.1097/00045415-200101000-00004, 11174910

[149] Beinart R Nazarian S. Effects of external electrical and magnetic fields on pacemakers and defibrillators. Circulation. (2013) 128:2799–809. doi: 10.1161/CIRCULATIONAHA.113.005697, 24366589 PMC3908865

[150] Stevenson R Carrillo R Gimbel JR Herkert RM. 16-50: electronic article surveillance systems remain a potential threat to CIED patients. EP Europace. (2016) 18:i13–i. doi: 10.1093/europace/18.suppl_1.i13a

[151] Wu D Qiang R Chen J Seidman S Witters D Kainz W. Possible overexposure of pregnant women to emissions from a walk through metal detector. Phys Med Biol. (2007) 52:5735–48. doi: 10.1088/0031-9155/52/19/001, 17881797

[152] Li C Wu T. "Numerical evaluation of exposure to the electromagnetic fields of an electronic article surveillance system with postured infant model". In: 2014 International Symposium on Electromagnetic Compatibility, Tokyo (2014). p. 206–9.

[153] Li C Wu T. Dosimetry for infant exposures to electronic article surveillance system: posture, physical dimension and anatomy. Bioelectromagnetics. (2015) 36:319–24. doi: 10.1002/bem.21901, 25756750

[154] Gandhi OP Kang G. Calculation of induced current densities for humans by magnetic fields from electronic article surveillance devices. Phys Med Biol. (2001) 46:2759–71. doi: 10.1088/0031-9155/46/11/301, 11720345

[155] Leitgeb N Niedermayr F Loos GDI. Impact of EAS systems on implanted cardiac pacemakers and defibrillators. J Electromagn Anal Appl. (2013) 5:67–73. doi: 10.4236/jemaa.2013.52011

[156] Takeda M Bannno Y Hirata H Okita Y Kimura M Sugiura T Electromagnetic field simulation around implantable cardiac pacemakers caused by EAS system [electronic article surveillance] 2004 Asia-Pacific Radio Science Conference, (2004) 444–446.

[157] Leitgeb N Niedermayr F Fuchs CHG. Impact of a radio frequency electronic article surveillance (EAS) system on active implants. J Electromagn Anal Appl. (2012) 4:353–7. doi: 10.4236/jemaa.2012.49049

[158] Ardeshirpour Y Cohen ED Seidman SJ Taddese B Zaidi T Bassen H. Effect of direct voltage induction by low-frequency security systems on neurostimulator lead. Bioelectromagnetics. (2024) 45:70–81. doi: 10.1002/bem.22485, 37870271

[159] Cecil S Schmid G. Computational Estimation of Personal Exposure against Electromagnetic fields Emitted by typical RFID Applications at 125 kHz and 13.56 MHz. Vienna, Austria: First International EURASIP Workshop on RFID Technology (2007). p. 29–30.

[160] Schneeweiss P Hirtl R Schmid G. Nonsinusoidal in situ electric field caused by magnetic deactivator device for EAS labels—assessment of field strength inside a detailed anatomical hand model. J Radiol Prot. (2023) 43:011514. doi: 10.1088/1361-6498/acb955 36745918

[161] Schmid G Schneeweiss P Hirtl R Jhala T Samaras T. Numerical assessment of induced electric fields in a worker's hand with commonly used metallic implants under exposure to low frequency magnetic fields. J Radiol Prot. (2024) 44:031507. doi: 10.1088/1361-6498/ad66dc, 39047759

[162] Floderus B Stenlund C Carlgren F. Occupational exposures to high frequency electromagnetic fields in the intermediate range ( >300 Hz–10 MHz). Bioelectromagnetics. (2002) 23:568–77. doi: 10.1002/bem.10050, 12395411

[163] Chiu RG Onyewuenyi A McGuire LS Mehta AI. Neurosurgical implants and interactions of airport metal detectors: a review. J Clin Neurosci. (2020) 77:254–7. doi: 10.1016/j.jocn.2020.05.012, 32402614

[164] Panagopoulos DJ Chrousos GP. Shielding methods and products against man-made electromagnetic fields: protection versus risk. Sci Total Environ. (2019) 667:255–62. doi: 10.1016/j.scitotenv.2019.02.344, 30831365

[165] Vaverka F Smetana M Gombarska D Psenakova Z. Investigation of microwave electromagnetic fields in open and shielded areas and their possible effects on biological structure. Sensors. (2023) 23:2351. doi: 10.3390/s23042351, 36850949 PMC9961501

[166] Constantinescu C Pacurar C Andreica S Gliga M Grindei L Rapolti L . Development and optimization of antennas for 860–960 MHz RFID applications and their impact on the human body. Technologies. (2026) 14:51. doi: 10.3390/technologies14010051

[167] Basu P Packer CJ Himstedt J. Detection of orthopaedic implants by airport metal detectors. J Bone Joint Surg Br Vol. (1997) 79-B:388–9. doi: 10.1302/0301-620x.79b3.0790388, 9180315

[168] Gottsauner-Wolf F Grohs JG. Detection of Orthopaedic prostheses at airport security checks. J Bone Joint Surg Br Vol. (1997) 79:385–7. doi: 10.1302/0301-620x.79b3.7604, 9180314

[169] McIvor ME Sridhar S. Interactions between cardiac pacemakers and antishoplifting security systems. N Engl J Med. (1998) 339:1394–5. doi: 10.1056/NEJM199811053391911, 9841304

[170] Ramirez MA Rodriguez EK Zurakowski D Richardson LC. Detection of orthopaedic implants in vivo by enhanced-sensitivity, walk-through metal detectors. J Bone Joint Surg Am. (2007) 89:742–6. doi: 10.2106/jbjs.F.00570, 17403795

[171] Abbassian A Datla B Brooks RA. Detection of orthopaedic implants by airport metal detectors. Ann R Coll Surg Engl. (2007) 89:285–7. doi: 10.1308/003588407x179026, 17394716 PMC1964703

[172] Koneru JN Dumitru I Easley AR Jr. Electromagnetic interference from electronic article surveillance system in a patient with a biventricular ICD and a left ventricular assist device. Pacing Clin Electrophysiol. (2011) 34:244–6. doi: 10.1111/j.1540-8159.2010.02842.x, 20727104

[173] Chinwalla F Grevitt MP. Detection of modern spinal implants by airport metal detectors. Spine (Phila Pa 1976). (2012) 37:2011–6. doi: 10.1097/BRS.0b013e31825aeccf, 22543252

[174] Chan JY Mani SB Williams PN O'Malley MJ Levine DS Roberts MM . Detection of in vivo foot and ankle implants by walkthrough metal detectors. Foot Ankle Int. (2014) 35:789–95. doi: 10.1177/1071100714534655 24845551

[175] Schneeweiss P Hirtl R Jhala T Ueberbacher R Cecil S Becker C . Electric field strengths and current densities induced inside the body due to exposure to electronic article surveillance (EAS) system antennas compared to ICNIRP’S exposure limits. J Radiol Prot. (2026) 46:021501. doi: 10.1088/1361-6498/ae553b 41861412

[176] ICNIRP. ICNIRP statement--health issues associated with millimeter wave whole body imaging technology. Health Phys. (2012) 102:81–2. doi: 10.1097/HP.0b013e31823a1278, 22134081

